# Multi-ancestry genome-wide association analyses incorporating SNP-by-psychosocial interactions identify novel loci for serum lipids

**DOI:** 10.1038/s41398-025-03418-z

**Published:** 2025-06-20

**Authors:** Amy R. Bentley, Michael R. Brown, Solomon K. Musani, Karen L. Schwander, Thomas W. Winkler, Mario Sims, Tuomas O. Kilpeläinen, Hugues Aschard, Traci M. Bartz, Lawrence F. Bielak, Jin-Fang Chai, Kumaraswamy Naidu Chitrala, Nora Franceschini, Mariaelisa Graff, Xiuqing Guo, Fernando P. Hartwig, Andrea R.V.R. Horimoto, Elise Lim, Yongmei Liu, Alisa K. Manning, Ilja M. Nolte, Raymond Noordam, Melissa A. Richard, Albert V. Smith, Yun Ju Sung, Dina Vojinovic, Rujia Wang, Yujie Wang, Mary F. Feitosa, Sarah E. Harris, Leo-Pekka Lyytikäinen, Giorgio Pistis, Rainer Rauramaa, Peter J. van der Most, Erin Ware, Stefan Weiss, Wanqing Wen, Lisa R. Yanek, Dan E. Arking, Donna K. Arnett, Christie Ballantyne, Eric Boerwinkle, Yii-Der Ida Chen, Martha L. Daviglus, Lisa de las Fuentes, Paul S. de Vries, Joseph A. C. Delaney, Amanda M. Fretts, Lynette Ekunwe, Jessica D. Faul, Linda C. Gallo, Sami Heikkinen, Georg Homuth, M. Arfan Ikram, Carmen R. Isasi, Jost Bruno Jonas, Liisa Keltikangas-Järvinen, Pirjo Komulainen, Aldi T. Kraja, Jose E. Krieger, Lenore Launer, Lifelines Cohort Study, Raul Aguirre-Gamboa, Patrick Deelen, Lude Franke, Jan A. Kuivenhoven, Esteban A. Lopera Maya, Ilja M. Nolte, Serena Sanna, Harold Snieder, Morris A. Swertz, Peter M. Visscher, Judith M. Vonk, Cisca Wijmenga, Naomi Wray, Jianjun Liu, Kurt Lohman, Annemarie I. Luik, Ani W. Manichaikul, Pedro Marques-Vidal, Yuri Milaneschi, Stanford E. Mwasongwe, Jeffrey R. O’Connell, Kenneth Rice, Stephen S. Rich, Pamela J. Schreiner, Lars Schwettmann, James M. Shikany, Xiao-ou Shu, Jennifer A. Smith, Harold Snieder, Nona Sotoodehnia, E. Shyong Tai, Kent D. Taylor, Lesley Tinker, Michael Y. Tsai, André G. Uitterlinden, Cornelia M. van Duijn, Diana van Heemst, Melanie Waldenberger, Robert B. Wallace, Hwee-Lin Wee, David R. Weir, Wen-Bin Wei, Ko Willems van Dijk, Gregory Wilson, Jie Yao, Kristin L. Young, Xiaoyu Zhang, Wei Zhao, Xiaofeng Zhu, Alan B. Zonderman, Ian J. Deary, Christian Gieger, Hans Jörgen Grabe, Timo A. Lakka, Terho Lehtimäki, Albertine J. Oldehinkel, Martin Preisig, Ya-Xing Wang, Wei Zheng, Michele K. Evans, Michael Province, James Gauderman, Vilmundur Gudnason, Catharina A. Hartman, Bernardo L. Horta, Sharon L. R. Kardia, Charles Kooperberg, Ching-Ti Liu, Dennis O. Mook-Kanamori, Brenda WJH Penninx, Alexandre C. Pereira, Patricia A. Peyser, Bruce M. Psaty, Jerome I. Rotter, Xueling Sim, Kari E. North, Dabeeru C. Rao, Laura Bierut, Clint L. Miller, Alanna C. Morrison, Charles N. Rotimi, Myriam Fornage, Ervin R. Fox

**Affiliations:** 1 Center for Research on Genomics and Global Health, National Human Genome Research Institute, Bethesda, MD 20892 USA; 2 Department of Epidemiology, School of Public Health, The University of Texas Health Science Center at Houston, Houston, TX 77030 USA; 3 MerexAgRight Farm, Ltd., Kitale, Kenya; 4 Division of Statistical Genomics, Washington University, St. Louis, MO USA; 5 Department of Epidemiology, University of Regensburg, Regensburg, 93051 Germany; 6 Department of Social Medicine, Population and Public Health, University of California, Riverside School of Medicine, Riverside, CA 92521 USA; 7 Novo Nordisk Foundation Center for Basic Metabolic Research, University of Copenhagen, Copenhagen, DK-2200 Denmark; 8 The Novo Nordisk Foundation Center for Genomic Mechanisms of Disease, Broad Institute of MIT and Harvard, Cambridge, MA 02142 USA; 9 Institut Pasteur, Université Paris Cité, Department of Computational Biology, F-75015 Paris, France; 10 Department of Epidemiology, Harvard School of Public Health, Boston, MA 02115 USA; 11 Department of Biostatistics, University of Washington, Seattle, WA 98101 USA; 12 Department of Epidemiology, School of Public Health, University of Michigan, Ann Arbor, MI 48109 USA; 13 Saw Swee Hock School of Public Health, National University of Singapore and National University Health System, Singapore, Singapore, 117549 Singapore; 14 Department of Engineering Technology, University of Houston, Sugar Land, TX 77479 USA; 15 Department of Epidemiology, University of North Carolina, Chapel Hill, NC 27516 USA; 16 Department of Pediatrics, The Institute for Translational Genomics and Population Sciences, The Lundquist Institute for Biomedical Innovation at Harbor-UCLA Medical Center, Torrance, CA 90502 USA; 17 Department of Epidemiology, Federal University of Pelotas, Pelotas, RS 96020-220 Brazil; 18 Heart Institute (InCor), University of São Paulo Medical School, São Paulo, SP 05403-900 Brazil; 19 Department of Biostatistics, Boston University, Boston, MA 02118 USA; 20 Department of Medicine, Division of Cardiology, Duke University School of Medicine, Durham, NC 27701-2047 USA; 21 Clinical and Translational Epidemiology Unit, Massachusetts General Hospital, Boston, MA 02114 USA; 22 Department of Medicine, Harvard Medical School, Boston, MA 02115 USA; 23 Department of Epidemiology, University of Groningen, University Medical Center Groningen, Groningen, 9713 GZ The Netherlands; 24 Department of Internal Medicine, Section of Gerontology and Geriatrics, Leiden University Medical Center, Leiden, 2333ZA the Netherlands; 25 McGovern Medical School, University of Texas Health Science Center at Houston, Houston, TX 77030 USA; 26 Department of Pediatrics, Baylor College of Medicine, Houston, TX 77030 USA; 27 Department of Biostatistics, School of Public Health, University of Michigan, Ann Arbor, MI 48109 USA; 28 Division of Biostatistics, Washington University, St. Louis, MO 63110 USA; 29 Department of Epidemiology, Erasmus MC University Medical Center, Rotterdam, the Netherlands; 30 Department of Biomedical Data Sciences, Leiden University Medical Center, Leiden, 2300 RC The Netherlands; 31 IQVIA, 1101 CT Amsterdam, the Netherlands; 32 Social, Genetic, and Developmental Psychiatry Centre, Institute of Psychiatry, Psychology and Neuroscience, King’s College London, London, UK; 33 Lothian Birth Cohorts, Department of Psychology, The University of Edinburgh, Edinburgh, EH8 9JZ UK; 34 Department of Clinical Chemistry, Fimlab Laboratories, Tampere, 33520 Finland; 35 Department of Clinical Chemistry, Finnish Cardiovascular Research Center - Tampere, Faculty of Medicine and Health Technology, Tampere University, Tampere, 33014 Finland; 36 Department of Psychiatry, Lausanne University Hospital and University of Lausanne, Prilly, Vaud 1008 Switzerland; 37 Foundation for Research in Health Exercise and Nutrition, Kuopio Research Institute of Exercise Medicine, Kuopio, 70100 Finland; 38 Survey Research Center, Institute for Social Research, University of Michigan, Ann Arbor, MI 48104 USA; 39 Interfaculty Institute of Genetics and Functional Genomics, University Medicine Greifswald, Greifswald, 17489 Germany; 40 German Center for Cardiovascular Research (DZHK), partner site Greifswald, Greifswald, 17475 Germany; 41 Division of Epidemiology, Vanderbilt University Medical Center, Nashville, TN 37203 USA; 42 Department of Medicine, Johns Hopkins University School of Medicine, Baltimore, MD 21287 USA; 43 McKusick-Nathans Institute, Department of Genetic Medicine, Johns Hopkins University School of Medicine, Baltimore, MD 21287 USA; 44 Department of Epidemiology and Biostatistics, Arnold School of Public Health, University of South Carolina, Columbia, SC 29169 USA; 45 Section of Cardiovascular Research, Baylor College of Medicine, Houston, TX 77030 USA; 46 Houston Methodist Debakey Heart and Vascular Center, Houston, TX 77030 USA; 47 Human Genome Sequencing Center, Baylor College of Medicine, Houston, TX 77030 USA; 48 Institute for Minority Health Research, University of Illinois Chicago, Chicago, IL 60612 USA; 49 Center for Biostatistics and Data Science, Institute for Informatics, Data Science, and Biostatistics (I2BD), Washington University School of Medicine, St. Louis, MO 63110 USA; 50 Division of Cardiology, Washington University, St. Louis, MO 63110 USA; 51 Department of Epidemiology, University of Washington, Seattle, WA USA; 52 Department of Medicine, University of Washington, Seattle, WA USA; 53 Cardiovascular Health Research Unit, University of Washington, Seattle, WA USA; 54 College of Pharmacy, University of Manitoba, Winnipeg, MB R3E 0T5 Canada; 55 Department of Medicine, University of Mississippi Medical Center, Jackson, MS 39213 USA; 56 Department of Psychology, San Diego State University, San Diego, CA USA; 57 Institute of Biomedicine, School of Medicine, University of Eastern Finland, Kuopio Campus, Kuopio, 70100 Finland; 58 Department of Epidemiology and Population Health, Albert Einstein College of Medicine, Bronx, NY USA; 59 Institute of Molecular and Clinical Ophthalmology Basel IOB, Basel, Switzerland; 60 Beijing Institute of Ophthalmology, Beijing Tongren Eye Center, Beijing Ophthalmology and Visual Science Key Lab, Beijing Tongren Hospital, Capital Medical University, Beijing, China; 61 Department of Psychology and Logopedics, University of Helsinki, Helsinki, 00014 Finland; 62 University of Mississippi Medical Center, Jackson, MS 39213 USA; 63 Laboratory of Epidemiology and Population Sciences, National Institute on Aging, National Institutes of Health, Baltimore, MD 21224 USA; 64 Genome Institute of Singapore, Agency for Science, Technology and Research, Singapore, Singapore, 138672 Singapore; 65 Department of Medicine, Duke Molecular Physiology Institute Duke University School of Medicine, Durham, NC 27701-2047 USA; 66 Center for Public Health Genomics, University of Virginia, Charlottesville, VA 22908 USA; 67 Department of Public Health Sciences, University of Virginia, Charlottesville, VA USA; 68 Department of Biochemistry and Molecular Genetics, University of Virginia, Charlottesville, VA USA; 69 Department of Medicine, Internal Medicine, Lausanne University Hospital and University of Lausanne, Rue du Bugnon 46, CH- 1011 Lausanne, Switzerland; 70 Department of Psychiatry, Amsterdam Neuroscience and Amsterdam Public Health Research Institute, Amsterdam UMC, Vrije Universiteit Amsterdam, Amsterdam, 1081 HJ the Netherlands; 71 Veritas Management Group, Inc., Alpharetta, GA 30072 USA; 72 Division of Endocrinology, Diabetes, and Nutrition, University of Maryland School of Medicine, Baltimore, MD USA; 73 Division of Epidemiology and Community Health, University of Minnesota, Minneapolis, MN USA; 74 Department of Health Services Research, School of Medicine and Health Services, Carl von Ossietzky Universität Oldenburg, Oldenburg, 26111 Germany; 75 Institute for Health Economics and Health Care Management, Helmholtz Zentrum München, German Research Center for Environmental Health, Neuherberg, 85764 Germany; 76 Division of Preventive Medicine, Heersink School of Medicine, University of Alabama at Birmingham, Birmingham, AL 35205 USA; 77 Cardiovascular Health Research Unit, Division of Cardiology, University of Washington, Seattle, Washington, 98195 USA; 78 Yong Loo Lin School of Medicine, National University of Singapore and National University Health System, Singapore, Singapore, 119228 Singapore; 79 Department of Pediatrics, LA Biomed at Harbor-UCLA Medical Center, Torrance, CA 90502 USA; 80 Public Health Sciences, Fred Hutchinson Cancer Center, Seattle, WA 98109 USA; 81 Laboratory Medicine and Pathology, University of Minnesota, Minneapolis, MN 55455 USA; 82 Department of Internal Medicine, Erasmus MC, University Medical Center, Rotterdam, The Netherlands; 83 Nuffield Department of Population Health, University of Oxford, Oxford, UK; 84 Research Unit Molecular Epidemiology, Institute of Epidemiology, Helmholtz Zentrum München, German Research Center for Environmental Health, Neuherberg, 85764 Germany; 85 Department of Epidemiology, University of Iowa College of Public Health, Iowa City, IA USA; 86 Beijing Tongren Eye Center, Beijing Key Laboratory of Intraocular Tumor Diagnosis and Treatment, Beijing Ophthalmology and Visual Sciences Key Laboratory, Beijing Tongren Hospital, Capital Medical University, Beijing, China; 87 Department of Human Genetics, Leiden University Medical Center, Leiden, 2333ZA The Netherlands; 88 Department of Internal Medicine, Division of Endocrinology, Leiden University Medical Center, Leiden, 2333ZA The Netherlands; 89 School of Public Health, Jackson State University, Jackson, MS 39213 USA; 90 Department of Population and Quantitative Health Sciences, Case Western Reserve University, Cleveland, OH 44106 USA; 91 Institute of Epidemiology, Helmholtz Zentrum München, German Research Center for Environmental Health, Neuherberg, 85764 Germany; 92 German Center for Diabetes Research (DZD), Partner München-Neuherberg, Neuherberg, Germany; 93 German Centre for Neurodegenerative Diseases (DZNE), Site Rostock/Greifswald, Greifswald, 17475 Germany; 94 Department of Psychiatry and Psychotherapy, University Medicine Greifswald, Greifswald, 17489 Germany; 95 Department of Clinical Physiology and Nuclear Medicine, Kuopio University Hospital, Kuopio, Finland; 96 Department of Psychiatry, University of Groningen, University Medical Center Groningen, Groningen, 9713 GZ the Netherlands; 97 Division of Biostatistics, Department of Population and Public Health Sciences, University of Southern California, Los Angeles, CA 90032 USA; 98 Icelandic Heart Association, Icelandic Heart Association, Kópavogur, 201 Iceland; 99 Faculty of Medicine, University of Iceland, Reykjavik, Iceland; 100 Postgraduate Program in Epidemiology, Federal University of Pelotas, Pelotas, RS 96020-220 Brazil; 101 Department of Clinical Epidemiology, Leiden University Medical Center, Leiden, 2333ZA the Netherlands; 102 Department of Public Health and Primary Care, Leiden University Medical Center, Leiden, 2333ZA the Netherlands; 103 Cardiovascular Health Research Unit, Departments of Medicine, Epidemiology, and Health Systems and Population Health, University of Washington, Seattle, USA; 104 Department of Psychiatry, Washington University School of Medicine, St. Louis, MO 63110 USA; 105 Department of Genetics, University of Groningen, University Medical Center Groningen, Groningen, The Netherlands; 106 Department of Pediatrics, University of Groningen, University Medical Center Groningen, Groningen, The Netherlands; 107 Department of Epidemiology, University of Groningen, University Medical Center Groningen, Groningen, The Netherlands; 108 Institute for Molecular Bioscience, The University of Queensland, Brisbane, QLD Australia

**Keywords:** Genomics, Depression

## Abstract

Serum lipid levels, which are influenced by both genetic and environmental factors, are key determinants of cardiometabolic health and are influenced by both genetic and environmental factors. Improving our understanding of their underlying biological mechanisms can have important public health and therapeutic implications. Although psychosocial factors, including depression, anxiety, and perceived social support, are associated with serum lipid levels, it is unknown if they modify the effect of genetic loci that influence lipids. We conducted a genome-wide gene-by-psychosocial factor interaction (G×Psy) study in up to 133,157 individuals to evaluate if G×Psy influences serum lipid levels. We conducted a two-stage meta-analysis of G×Psy using both a one-degree of freedom (1df) interaction test and a joint 2df test of the main and interaction effects. In Stage 1, we performed G×Psy analyses on up to 77,413 individuals and promising associations (*P* < 10^−5^) were evaluated in up to 55,744 independent samples in Stage 2. Significant findings (*P* < 5 × 10^−8^) were identified based on meta-analyses of the two stages. There were 10,230 variants from 120 loci significantly associated with serum lipids. We identified novel associations for variants in four loci using the 1df test of interaction, and five additional loci using the 2df joint test that were independent of known lipid loci. Of these 9 loci, 7 could not have been detected without modeling the interaction as there was no evidence of association in a standard GWAS model. The genetic diversity of included samples was key in identifying these novel loci: four of the lead variants displayed very low frequency in European ancestry populations. Functional annotation highlighted promising loci for further experimental follow-up, particularly rs73597733 (*MACROD2*), rs59808825 (*GRAMD1B*), and rs11702544 (*RRP1B*). Notably, one of the genes in identified loci (*RRP1B*) was found to be a target of the approved drug Atenolol suggesting potential for drug repurposing. Overall, our findings suggest that taking interaction between genetic variants and psychosocial factors into account and including genetically diverse populations can lead to novel discoveries for serum lipids.

## Introduction

The concentrations of key serum lipids, such as high-density lipoprotein cholesterol (HDLC), low-density lipoprotein cholesterol (LDLC), and triglycerides (TG) are routinely assessed to determine an individual’s cardiometabolic clinical risk profile, and to guide drug therapy (e.g., statins) aiming to reduce the morbidity and mortality associated with diseases such as coronary artery disease, stroke, and type 2 diabetes. Serum lipid levels are known to be influenced both by lifestyle, including diet, physical activity, smoking, and alcohol consumption, as well as genetic factors, with over 700 lipids loci identified using genome-wide association studies (GWAS) [1–3]. Although the importance of both genetic and lifestyle factors is well-established, the interplay between these two factors on serum lipid levels is less well understood. The Gene-Lifestyle Interactions Working Group, under the aegis of the Cohorts for Heart and Aging Research in Genomic Epidemiology (CHARGE) Consortium [4], has developed a framework for studying gene-lifestyle interactions for cardiometabolic traits in large, multi-ancestry meta-analyses [5]. This strategy has facilitated the discovery of novel lipids loci in studies accounting for interactions with smoking [6], physical activity [7], alcohol consumption [8], educational attainment [9], and sleep duration [10], suggesting that these lifestyle factors may indeed modulate genetic effects on serum lipids. The loci identified in these efforts could potentially explain how lifestyle exposures can contribute to disturbances in lipid levels.

Psychosocial factors, especially depression, contribute to the pathogenesis of cardiovascular diseases (e.g., myocardial infarction) and increased mortality in patients with coronary heart disease [11–13], and Mendelian Randomization (MR) analysis suggests that this association is causal [14]. Depression and depressive symptoms are associated with serum lipid concentrations [13, 15], the plasma lipidome [16, 17], and lipid metabolism, with distinct metabolic signatures associated with various symptom dimensions [18]. *APOE* alleles associated with serum lipids have also been associated with anxiety and depression [19, 20]. Some evidence from MR analyses suggests that depression increases TG and decreases HDLC [21]. Serum lipids may also mediate the association between depression and cardiovascular disease. The association between depression and coronary artery disease was attenuated when an MR analysis was adjusted for serum lipids [14]. Similarly, in one study, nearly a third of the association of depression with arterial stiffness, a key intermediary of major cardiovascular events, was found to be mediated by metabolic syndrome, particularly hypertriglyceridemia among men [22]. Low social support has been associated with high cholesterol in a nationally representative US nonelderly population [23] and with high cholesterol, LDL, and non-HDL cholesterol among type 2 diabetics and their families [24]. Both anxiety and depression have been associated with elevated triglycerides [25]. Proposed mechanisms through which psychosocial factors and serum lipids may influence each other include high dietary intake of saturated fat and cholesterol, gut dysbiosis, the hypothalamic-pituitary-adrenal axis, and neuroinflammation [26–28]. Both direct and indirect mechanisms, such as psychosocial factor-associated changes in lifestyle or medication use, are plausible. This may confound interaction effects. Importantly, there is evidence for a genetic contribution to some of these psychosocial factors, particularly depression [29–33].

In this study, we assess how incorporating interaction between genetic variants and psychosocial factors (depressive symptoms, anxiety symptoms, and low social support) helps identify lipid loci missed by standard marginal genetic effect GWAS. To maximize the transferability of our results and to address known gaps in the field, we prioritized the inclusion of diverse population groups, as ancestry can influence both genetic (e.g. frequency of variants, linkage disequilibrium around associated signals) and psychosocial factors (e.g. presence of stigma, availability of healthcare access). We conducted multi-ancestry meta-analyses of genome-wide variant × psychosocial factor interaction (G×Psy) studies on serum lipids in up to 133,157 individuals.

## Results

**Fig. 1:**
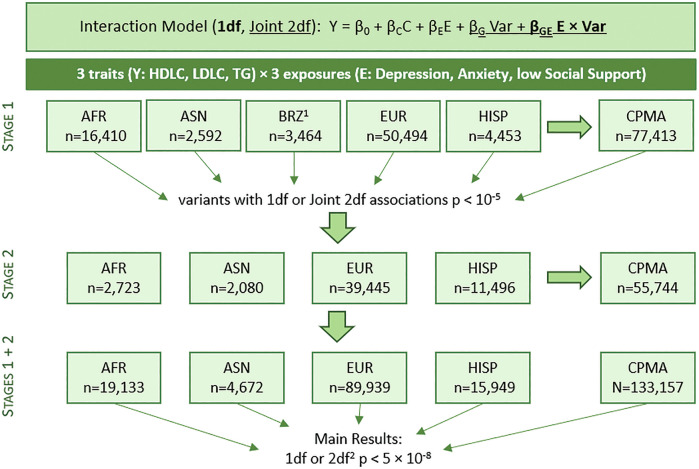
Study design. *African ancestry (AFR), Asian ancestry (ASN), Brazilian (BRZ), European ancestry (EUR), Hispanic (HISP), cross-population meta-analysis (CPMA)1 Brazilian samples were only available in Stage 1, so a Stage 1* *+* *2 meta-analysis of BRZ was not possible. These samples are include in the cross-population Stage 1* *+* *2 meta-analysis; 2 As the 2df results joinly measure the variant's main and interaction effects, our main results only include those 2df findings that are also more than 500 kb from known lipids loci*.

In this study of psychosocial factors and serum lipids, we meta-analyzed data on up to 133,157 individuals from 50 genome-wide interaction studies using a two-stage study design (Fig. 1; Study Details Supplementary Tables S1-2; Supplementary Note). Sample sizes and descriptive statistics of the studies participating in Stages 1 and 2 analyses are summarized in Supplementary Table 2. Study participants included European ancestry (EUR; 67.5%; *n* = 89,939), African ancestry (AFR; 14.4%; *n* = 19,133), Hispanic/Latino (HISP; 12.0%; *n* = 15,949), Asian ancestry (ASN; 3.5%; *n* = 4672), and Brazilian individuals (BRZ; 2.6%; *n* = 3464; see Methods for further details regarding selection of groups and population descriptors). All psychosocial factors were coded as binary variables. On average, 17.5% of Stage 1 participants were reported to have elevated depressive symptoms (DEPR) and 24.2% had elevated anxiety symptoms [ANXT], based on standard cutpoints, with a similar distribution among Stage 2 participants (15.8% [DEPR] and 24.3% [ANXT]). Low social support (SOCS) was defined as the lowest quartile of the distribution (Methods). Fewer studies had data on SOCS (*n* = 23) and ANXT (*n* = 21) than DEPR (*n* = 50; harmonization of psychosocial factors is described in Supplementary Table 3).

**Fig. 2:**
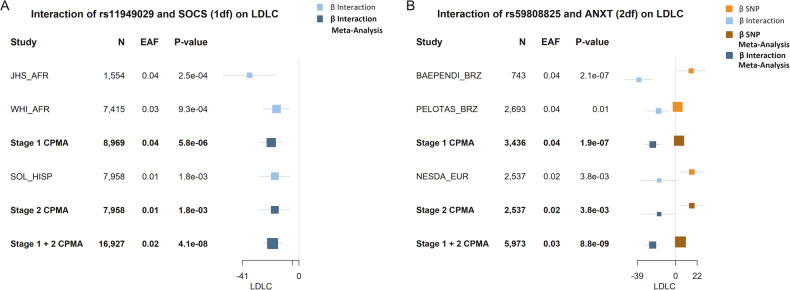
Forest Plots of Key Findings. Forest plot showing all studies contributing data on an interaction of **A**. rs11949029 × social support (SOCS) on LDLC using a 1df test of the interaction term (this interaction was also statistically significant using the 2df joint test of the main effect and interaction, shown in Supplementary Figure 4D); and **B**. rs59808825 × anxiety symptoms (ANXT) on LDLC. Box size represents the precision of the estimate, with larger boxes shown for results with lower variance. *Abbreviations: African ancestry (AFR), Brazilian (BRZ), Effect Allele Frequency (EAF), European Ancestry (EUR), Hispanic (HISP), cross-population meta-analysis (CPMA)*.

In Stage 1 population-specific and cross-population meta-analyses (CPMA), we identified 15,774 variants that met our selection criteria of *P* < 10^−5^ for either the 1 degree of freedom interaction test (1df) or the 2df joint test of the main and interaction effects (2df; Fig. 1). These variants were carried forward for further analysis in Stage 2 samples. In the meta-analysis of stages 1 and 2, there were 10,230 variants from 120 loci that were significantly associated in at least one model using either statistical test (*P*_1df_ or *P*_2df_ < 5 × 10^−8^; Supplementary Table 4). We found seven variants in four loci that were associated with serum lipids with a genome-wide significant p-value for the 1df test of interaction (Table 1). For instance, among those reporting low social support, the A allele of rs11949029 was associated with a much lower LDLC concentration than among those who did not (β_Interactio_*n* = −19.2, SE 3.5 mg/dL, *P*_1df_ = 4.1 × 10^−8^; Fig. 2A). Thus, among those with low social support, this allele was associated with 12.6 mg/dL lower LDLC, but 6.6 mg/dL higher LDLC among those not reporting low social support. In meta-analyses that did not include a multiplicative term (i.e. a standard GWAS model; available only for stage 1 studies), no association of the variant with LDLC was observed (*P* = 0.69), even after adjustment for SOCS (*P* = 0.75).

**Table 1:** Genome-wide variants significantly associated with serum lipids using a 1 df test of interaction in Meta-Analysis of Stages 1 & 2.

rsid Index Variant *Nearest Gene*^*a*^	Chr:BP (GRCh37	Tested Allele: Freq	Main Effect	SE	Interaction Effect	SE	P_1df_^b^	P_2df_^b^	Population, Sample Size	Lipid^c^	Psychosocial Factor
rs11949029 *RNU4-73P*	5:163470425	A:0.02	6.6	2.2	-19.2	3.5	**4.1E-08**	**2.4E-08**	CPMA, *n* = 16,927	LDLC	SOCS
rs59808825 *GRAMD1B*	11:123152496	C:0.03	5.0	2.1	-22.9	4.2	**4.3E-08**	**8.8E-09**	CPMA, *n* = 5,973	LDLC	ANXT
rs61248562 *RP11-643A5.2*	15:54249794	I^d^:0.02	−0.024	0.015	0.14	0.025	**5.2E-09**	6.9E-08	EUR, *n* = 15,052	HDLC	DEPR
rs73597733 *MACROD2*	20:15941137	A:0.04	−0.015	0.010	0.14	0.025	**8.4E-09**	**1.0E-08**	AFR, *n* = 11,234	HDLC	DEPR

^a^Nearest gene determined based on ANNOVAR annotations implemented in FUMA. While the “Nearest Gene” is listed for simplicity, we acknowledge that the nearest gene may not be of functional relevance for the given variant.

^b^Bolded values indicate *P* < 5 × 10^−8^.

^c^Analyses were conducted on natural log-transformed HDLC and TG values, while LDLC was untransformed (all original values in mg/dL).

^d^Insertion.

A significant association using the 2df joint test of the main effect and interaction can represent the main effect of a variant, its interaction with the exposure, or both. To exclude associations driven primarily by the main effect, we considered as previously unidentified only those variants with *P*_2df_ < 5 × 10^−8^ that were independent of known loci (defined as ± 500 kb from the 95% credible sets reported in Graham *et al* [1] or variants reported in other major publications [34–39]). There were 14 variants and 8 loci that were significantly associated with serum lipids using the 2df test and independent from known loci (Table 2). Six of these loci displayed nominal significance for an interaction effect (*P*_1df_ < 0.05). This includes three of the four loci that were genome-wide significant using the 1df test, with the remaining two near this threshold. Among these is rs59808825 (*GRAMD1B* [nearest gene]), for which the main effect of the C allele on LDLC was positive (β = 5.0, SE = 2.1 mg/dL) but an inverse interaction effect with ANXT (β_Interaction_ = −22.9, SE = 4.2 mg/dL), so that the total effect of the C allele among those reporting anxiety symptoms was negative (*P*_2df_ = 8.8 × 10^−9^; Fig. 2B). In the main effect only meta-analysis, no association between rs59808825 and LDLC was observed (*P* = 0.13). Of the 8 loci identified through the 2df test, two associations had *P*_1df_ ≥ 0.1, suggesting that the 2df test may reflect novel main effect associations (though we cannot exclude that G×Psy interactions may contribute to these findings but are undetectable at the current sample sizes). For instance, the joint 2df test was significant for rs34636484 (*CD96*) × SOCS on LDLC (P_2df_ = 3.1E-8), while the 1df test was not (P_1df_ = 0.27; Supplementary Figure 4C). rs11702544 (*RRP1B*) × DEPR on HDLC was also plausibly driven by a main effect (Supplementary Figure 4H). Importantly, both of these potential main effect associations were identified in the CPMA results, highlighting the importance of including diverse populations for novel discoveries.

**Table 2:** Genome-wide variants significantly associated with serum lipids using a 2df joint test of main effect and interaction in Meta-Analysis of Stages 1 & 2 and were independent of known lipids loci^a^.

rsid Index Variant *Nearest Gene*^b^	Chr:BP (GRCh37)	Tested Allele: Freq	Main Effect	SE	Interaction Effect	SE	P_1df_^c^	P_2df_^c^	Population, Sample Size	Lipid^d^	Psychosocial Factor
rs6730082 *RNU4-73P*	2:9877621	C:0.26	−0.0038	0.0040	−0.045	0.0096	3.1E-06	**2.5E-08**	AFR, *n* = 16,886	HDLC	DEPR
rs142378953 *AC090043.1*	3:1695235	G:0.01	0.039	0.032	0.24	0.060	6.0E-05	**4.9E-08**	CPMA, *n* = 18,911	TG	DEPR
rs34636484 *CD96*	3:111356092	G:0.03	-4.5	0.80	1.8	1.6	0.27	**3.1E-08**	CPMA, *n* = 42,162	LDLC	SOCS
rs11949029 *CTC-207P7.1*	5:163470425	A:0.02	6.6	2.2	−19.0	3.5	**4.1E-08**	**2.4E-08**	CPMA, *n* = 16,927	LDLC	SOCS
rs4562311 *NKAIN3*	8:63589187	G:0.22	0.013	0.014	−0.13	0.032	3.5E-05	**8.5E-09**	CPMA, *n* = 6,667	TG	ANXT
rs59808825 *GRAMD1B*	11:123152496	C:0.03	5.0	2.1	−22.9	4.2	**4.3E-08**	**8.8E-09**	CPMA, *n* = 5,973	LDLC	ANXT
rs73597733 *MACROD2*	20:15941137	A:0.04	-0.015	0.010	0.14	0.025	**8.4E-09**	**1.0E-08**	AFR, *n* = 11,234	HDLC	DEPR
rs11702544 *RRP1B*	21:45091861	C:0.39	0.0049	0.0010	0.0043	0.0027	0.1	**2.3E-08**	CPMA^e^/EUR, *n* = 100,182	HDLC	DEPR

^a^Listed variants were more than 500 KB from 95% Credible Sets reported in Graham et al. (10.1038/s41586-021-04064-3).

^b^Index variant is for the most statistically significant association in the region and Nearest Gene is determined based on ANNOVAR annotations implemented in FUMA. While the “Nearest Gene” is listed for simplicity, we acknowledge that the nearest gene may not be of functional relevance for the given variant.

^c^Bolded values indicate *P* < 5 × 10^−8^.

^d^Analyses were conducted on natural log-transformed HDLC and TG values, while LDLC was untransformed (all original values in mg/dL).

^e^The most statistically significant analysis (indicated) is displayed in table. Full results for all genome-wide significant associations given in Supplementary Table S4.

The inclusion of underrepresented population groups in this study also provided an advantage in identifying novel interaction associations, with associations observed at four lead loci at which no data from EUR studies were available because of a minor allele frequency < 0.01. For instance, an interaction of rs11949029 (*CTC-207P7.1*) and SOCS on LDLC was statistically significant for both the 1df test of interaction (Fig. 2A) and the 2df test of interaction and main effect (Supplementary Figure 4D) and was driven by data from the AFR and HISP populations. In this case, there was consistency of both the main and interaction associations across backgrounds. Such consistency was common among these lead findings; however, there were a few associations that were driven predominantly by one population, despite the availability of data for other groups. In the interaction between rs61248562 (*UNC13* *C*) and DEPR on HDLC, the observed association among EUR reached statistical significance (β = 0.14; SE = 0.025; *P*_1df_ = 5.2 × 10^−9^; Supplementary Figure 3C), yet this association was not seen in other populations despite a comparable number of samples with available data (EUR 15,052 vs. AFR 13,069 and HISP 15,977) and larger effect allele frequencies (EUR 0.02 vs. AFR 0.16 and HISP 0.07). As expected, the CPMA for this association was greatly reduced in statistical significance (*P*_1df_ = 1.5 × 10^−3^). Similarly, the rs73597733 (*MACROD2*) × DEPR interaction on HDLC in AFR (*P*_1df_ = 8.4 × 10^−9^) was not seen in HISP (*P*_1df_ = 0.43; Supplementary Figure 3D).

Of the 10 lead associations in 9 loci that reached genome-wide statistical significance in the meta-analyses of Stages 1 and 2 (for one locus there were significant associations in two population groups), 4 were significant in both stages (*P* < 0.05) while 6 were only significant in stage 1 (Supplementary Table 5). There were 16 variants in 9 loci that were considered as the novel associated variant set for annotation and follow-up: those associated with either *P*_1df_ < 5 × 10^−8^ (seven variants in four loci) or *P*_2df_ < 5 × 10^−8^ and independent of known lipids loci (14 variants in 8 loci; five variants in three loci overlapping in 1df and 2df findings). The novel associated variants were characterized using FUMA. As expected, most of the variants were annotated to be intronic (*n* = 10) or intergenic (*n* = 3; Supplementary Table 6). While a single signal was detected for most of of the described loci in Tables 1 and 2, the associated region on chromosome 21 (CPMA-HDL-DEPR) had three independent genomic signals at variants rs11702544, rs6518309, and rs9977076. Each of these variants is an eQTL for three genes in a variety of tissues, including whole blood: *PDXK*, *RRP1B*, and *HSF2BP* [40] (Supplementary Table 7).

We also evaluated 257 variants in LD with our lead variants (R^2^ ≥ 0.6 in 1000 Genomes, Phase 3 ALL; Supplementary Table 8). Evaluation of these variants in RegulomeDB identified 75 variants (29.2%) with functional prediction scores ≤ 3, indicating moderate to high potential for regulatory effects. Variants within the locus on chromosome 21 characterized by rs11702544, rs6518309, and rs9977076 (*RRP1B*) had the lowest RegulomeDB scores in this set: 1a (*n* = 1) and 1b (*n* = 6), which indicates that they are likely to affect transcription factor binding to the gene targets, in this case *HSF2BP*, *RRP1B*, or *LINC00313*. These variants were also tested in our data and nearly reached statistical significance for the 2df interaction with DEPR on HDLC (*P*_2df_ range 2.4 × 10^−6^ to 4.2 × 10^−7^), with similar effect sizes in all.

Next, we assessed the predicted chromatin state around our 16 novel associated variants using the minimum 15-core chromatin state models calculated across 127 tissue/cell types [41]. We identified histone chromatin markers in regions associated with strong transcription (*n* = 6; Supplementary Table 6). In the 257 variants in LD with our lead variants, there were histone chromatin markers consistent with active (*n* = 13) or flanking active (*n* = 21) transcription start sites, transcription at the 3′ or 5′ end (*n* = 7), or in regions associated with strong transcription (*n* = 50) (Supplementary Table 8). For most of our loci, significant chromatin interactions were detected between regions containing our variants and regions overlapping gene promoters (Supplementary Table 9); for instance, between the locus on chromosome 21 (lead variant rs11702544) and regions overlapping the promoter of multiple genes, including *PDXK*, *RR1BP*, and *HSF2BP*.

Finally, to explore the potential clinical relevance of our findings, we performed an integrated druggability analysis of identified genes, as previously described [42]. We queried high and medium priority candidate gene targets (identified by FUMA and OpenTargets) using the Drug-Gene Interaction database (DGIdb), which revealed 2 genes annotated as clinically actionable or members of the druggable genome (Supplementary Table 10). Several of these gene targets are implicated in ion transport (*NKAIN3*), vitamin metabolism (*PDXK*), and immune or viral response (*CD96*, *RRP1B*) pathways. We identified 1 gene, *RRP1B*, with a reported drug interaction. *RRP1B* was shown to interact with an FDA-approved drug, Atenolol, that has been evaluated in late-stage clinical trials using DrugBank and ClinicalTrials.gov databases (Supplementary Table 10). Atenolol is a well-established anti-hypertensive drug used to treat high blood pressure, heart failure, or angina in some patients. Together these results suggest a potential drug repurposing opportunity to intervene in a common pathway implicated in cardiometabolic disorders.

## Discussion

In this study, we investigated genome-wide variant-by-psychosocial factor interactions (G×Psy) in large, multi-ancestry meta-analyses of serum lipids. We identified nine novel lipid loci using this strategy, including four loci based on the 1df test of interaction and eight loci based on the 2df joint test of interaction and main effects (with three loci significantly associated using both strategies). Importantly, most of these associations could not have been identified in a standard GWAS that does not take interaction into account. Our inclusion of relatively large sample sizes representing diverse ancestries facilitated novel findings. Functional annotation highlights the promise of some of these identified loci for understanding the potential influence of psychosocial factors on serum lipids.

Both the 1df test of interaction and the 2df test of main effect and interaction identified statistically significant results for rs73597733 (intronic to *MACROD2*) × DEPR on HDLC, in which the main effect of the variant was near zero, with a large positive association among those with depressive symptoms. Intriguingly, an interaction between an intronic variant in *MACROD2* (not in LD with rs73597733) was previously found between thiazide diuretic use and HDLC [43]. Other intronic variants in *MACROD2* have been associated with the ceramides and sphingomyelins, suggesting a potential role in lipids pathways [44]. There is a large body of evidence for associations of intronic variants in *MACROD2* with complex psychosocial, neurological, and psychiatric traits, including: attention deficit hyperactivity disorder [45, 46], morningness (being a morning person) [47], risk-taking behavior [48], eating disorders [49], autism [50–52], and bipolar disorder [52, 53]. Infants with atypical neonatal neurobehavioral scores had differentially methylated CpG sites within the *MACROD2* gene [54]*. Macrod2* knockout mouse models displayed hyperactivity that became more pronounced with age [55]. Intronic variants in *MACROD2* have also been associated with measures of cognitive ability [56–58] and a variety of brain measurements [59–63]. Given the significant evidence for the involvement of this gene in a range of complex psychological and psychiatric phenotypes and a previous finding for an interactive effect on HDLC, our reported finding of an interaction between an intronic variant in *MACROD2* and DEPR on HDLC seems of particular interest and worthy of further investigation.

An association between rs59808825 (110 kb upstream of *GRAMD1B*) and ANXT on LDLC was *P* < 5 × 10^−8^ for both the 2df joint test of main effect and interaction and the 1df test of interaction, and no association was observed for this variant in an analysis without interaction modeled. *GRAMD1B* was identified as a locus for schizophrenia in multiple studies [64–69], a condition that has been linked with anxiety [70, 71]. The protein encoded by *GRAMD1B*, Gramd1b or Aster-B, has a role in cholesterol homeostasis, transporting accessible cholesterol from the plasma membrane to the endoplasmic reticulum [72, 73]. It was recently discovered that Aster proteins including Aster-B are key players in dietary lipid absorption in mice: the systemic absorption of dietary cholesterol was reduced by treatment with a small-molecule Aster inhibitor and mice without intestinal Aster proteins were protected from diet-induced hypercholesterolemia [74]. While further investigation is needed to propose a biological mechanism that might underlie the observed interaction between this variant and ANXT on LDLC, the known associations between nearby *GRAMD1B* with both complex psychiatric and psychological phenotypes and absorption of dietary lipid are intriguing.

We identified a 2df interaction of DEPR with variants on chromosome 21 (lead variant rs11702544 [*RRP1B*]) that appeared to represent a novel main effect of a common variant on HDLC. Interestingly, there was some evidence for an association of rs11702544 with HDLC using a standard GWAS model in the recent Global Lipids Genetics Consortium results (*P* = 2.2E-6) [1], consistent with the contribution of a main effect of this variant contributing to the 2df joint test of main effect and interaction. FUMA annotation identified 3 independent genomic loci in this region, each of which is an eQTL for *PDXK*, *RRP1B*, and *HSF2BP*. Each of the genes has been previously associated with risk of diseases for which serum lipids concentration is a key risk factor: *PDXK* and *RRP1B* with coronary artery disease [75] and *HSF2BP* with cardiovascular disease [76]. *PDXK* encodes a protein essential for the generation of the active form of Vitamin B_6_. *PDXK* mRNA levels in adipose tissue were strongly associated with adipogenic, lipid-droplet-related, and lipogenic genes, and administration of the active form of Vitamin B_6_ led to increased adipogenic markers in adipocyte precursor cells [77]. While the role for variants in this locus in HDLC concentration is not clear, they have been shown to affect *PDXK* expression, which could affect HDLC concentration through the expression of genes involved in lipogenesis. Our druggability analysis also identified *PDXK* as part of the druggable genome. *RRP1B* is a target gene that interacts with the beta-blocker drug Atenolol, which is sometimes used to treat hypertension and chronic angina.

We also identified an association using the 2df test rs34636484 (*CD96*) × SOCS on LDLC. The main effect appeared to contribute more to the association than the interaction at this locus, as the association was also apparent in a standard GWAS model in our data and the 1df test of interaction was not significant (*P* = 0.27). Based on these results, the association at rs34636484 appears to represent a novel main effect locus; however, this result should be interpreted with caution. The association of rs34636484 and LDLC was recently evaluated in the Global Lipids Genetics Consortium with a much larger sample size (*n* = 1,393,230 at this locus) and was not statistically significant (*P* = 0.029) [1].

Some of our significant associations were fairly consistent across studies within the same population group, but with no compelling evidence of association in other population groups, despite the availability of data. For instance, rs61248562 (*UNC13C*) × DEPR on HDLC was significant only among EUR, and not AFR or HISP in whom allele frequencies were higher and sample sizes were comparable. Similarly, an interaction of rs73597733 (*MACROD2*) × DEPR on HDLC in AFR was not seen in HISP at similar sample sizes (with a slightly lower allele frequency). It is unclear why these associations may differ by population group, but this phenomenon has been reported in previous gene-lifestyle interaction publications [6, 78, 79]. Differences in gene-lifestyle interactions across populations may arise from genomic factors, such as variations in linkage disequilibrium that lead to the tagging of different variants, as well as from lifestyle factors, such as differences in the measurement of or the experience of the psychosocial factor or in the behaviors or conditions associated with that psychosocial factor.

Psychosocial factors are complex traits that are associated with a variety of other factors, including some lifestyle exposures that we have previously evaluated using the same genome-wide interaction study approach. Overlap in the interaction results for this study and previous analyses for one of these associated lifestyle factors could be very informative for disentangling the mechanism underlying these statistical interactions. We compared our statistically significant findings with those that we have previously reported for genome-wide interactions of smoking [6], alcohol intake [8], physical activity [7], educational attainment [9], and sleep duration [10] on serum lipids; no overlap among the results was identified. If one of our loci were found to be associated with a psychosocial factor, that could provide additional context into the relationship between psychosocial factors and serum lipids. To explore this possibility, we evaluated recent GWAS for these traits [29, 33, 80–84], but did not identify any overlap with our loci of interest.

Some of the strengths of this study include the relatively large sample sizes for a study of psychosocial factors, with analyses including up to 133,157 individuals. Also notable was the particular attention to the inclusion of non-European ancestry individuals (reaching over 19,000 AFR and nearly 16,000 HISP, although the number of ASN and BRZ was smaller, <5000 per population). The sample sizes for the non-European ancestry groups, however, were relatively small in size, particularly in terms of the statiscial power needed for a gene-environment interaction study. We used a two-stage design with both a 1df test of interaction and a 2df joint test of main effect and interaction, an approach that is well-established for the study of gene-lifestyle interactions [6–10, 78, 79, 85, 86]. Our study also has some limitations. First, we had a smaller sample size for Stage 2, particularly for certain populations; as a result, the power for our two-stage approach was reduced. Second, despite our best efforts to harmonize psychosocial factors, the use of different instruments to measure these outcomes may have resulted in heterogeneity among studies, which would have reduced the power to identify lipids loci. In addition, these phenotypes themselves are quite complex and heterogeneous, and that complexity is not reflected in our categorization. Moreover, although our sample size is large for a study of lipids and psychosocial factors, it is not large enough to enable correction for multiple testing with adequate statistical power, and so its results need further validation. We did not have enough statistical power to usefully evaluate differences in these interactions by sex, which may prove to be of interest, as there are differences in the pathophysiology of cardiovascular disease by sex, and women experience a greater burden of depression [87]. Additionally, the association between TG and depressive symptoms has been shown to differ by sex, with men showing a stronger association [88], and low social support had a greater adverse effect on cardiovascular disease prevention among men than women [89]. Evaluating these interactions would require much greater sample sizes than were available in the current study. Although we have organized our contributing studies into population groups, there is likely to be meaningful heterogeneity within those groups in terms of relevant environmental background. For instance, the East Asian population group included individuals living in China as well as individuals with ancestry in China living in the United States. Information regarding neuropsychiatric medication use was not collected, though it is possible that use of these medications might directly or indirectly influence serum lipid levels [90]. In silico functional annotation and druggability analyses identified loci and candidate drug-gene interactions that are of interest for further follow-up; future experimental studies are needed to validate these findings.

In summary, we identified novel lipids loci in this large, multi-ancestry meta-analyses of genome-wide interaction studies of variants and psychosocial factors. Understanding these loci may help to disentangle the complex interplay between factors such as anxiety, depression, and low social support on serum lipids, a key biomarker of cardiometabolic risk.

## Materials and methods

### Study design

We adopted a two-stage study design (Fig. 1) that was implemented according to the Gene-Lifestyle Interactions Working Group of the CHARGE consortium [5]. We included men and women aged between 18 to 80 years of age with available data on lipids and psychosocial factors, and with genotype data imputed to the 1000 Genomes reference panel.

Stage 1 included 77,413 individuals in 31 study/population groups. Each study conducted genome-wide analyses (GWAS) incorporating a variant-by-psychosocial factor multiplicative interaction term. Centralized quality control was carried out, which was followed by a meta-analysis within and across five population groups: African ancestry (AFR), Asian ancestry (ASN), Brazilian (BRZ), European ancestry (EUR), and Hispanic (HISP). Variants that showed suggestive (*P* < 10^−5^) associations for either a 1df test of interaction or a 2df joint test of interaction and main effect were carried forward for evaluation in Stage 2. Stage 2 analyses included data on 55,744 individuals from 19 studies distributed in 4 population groups. As no BRZ samples were included in Stage 2, no population-specific Stage 1 + 2 meta-analysis was undertaken, though the BRZ samples were included in cross-population meta-analyses (CPMA). Analytical details (Supplementary Table S1) and descriptive statistics (Supplementary Table S2) of each participating study for Stages 1 and 2 are provided.

### Phenotypes and lifestyle variables studied

Analyses were conducted separately for three lipid parameters: HDLC, LDLC, and TG. HDLC and TG were directly assayed and natural log-transformed prior to analysis. LDLC was either directly assayed or derived using the Friedewald equation: LDLC = TG – HDLC – (TG / 5), if TG ≤ 400 mg/dL [91]. If a sample was drawn from an individual who had not been fasting for at least 8 h, then neither TG nor derived LDLC values were used. LDLC values were adjusted for lipid-lowering medication use (defined as the use of a statin or of any unspecified lipid-lowering medication after 1994, when statin usage became common). If LDLC was directly assayed, adjustment for lipid-lowering drugs was performed by dividing the LDLC value by 0.7. If LDLC was derived using the Friedewald equation, total cholesterol was first adjusted for lipid-lowering drug use (total cholesterol/0.8) before calculation of LDLC. No adjustments were made for any other lipid medication, nor were adjustments made to HDLC or triglycerides for medication use. For longitudinal studies where multiple lipid measurements were available, analysts selected the measurement with the largest sample size for analysis.

The three psychosocial variables (elevated depressive symptoms [DEPR], low social support [SOCS], and elevated anxiety symptoms [ANXT]) were measured within each cohort using validated screening questionnaires and coded as binary (yes/no) variables. A standard cut point was used for DEPR and ANXT, and SOCS was defined based on the lowest quartile of perceived social support. Further details regarding the instruments used within each study are given (Supplementary Table 3). Where multiple measurements of psychosocial factors were available, we used the questionnaire administered concomitantly with the measurement of serum lipids.

### Genotyping and imputation

To harmonize data across studies, all studies imputed to 1000 Genomes data. Details on genotyping and imputation for each of the included studies are given in Supplementary Table 1. Most studies used Affymetrix (Santa Clara, CA, USA) or Illumina (San Diego, CA, USA) arrays and imputed to the cosmopolitan reference panel of the 1000 Genomes Project Phase I Integrated Release Version 3 Haplotypes. Prior to analysis, studies excluded all variants with minor allele frequency <0.01 or those that mapped to the X and Y chromosomes or the mitochondria.

### Study-level genome-wide analysis

Each cohort participating in Stage 1 analysis regressed serum lipids (Y) on the variant (G), psychosocial factor (E), and their interaction (G×E), with adjustment for covariates (C) including age, sex, principal components, and study-specific variables (listed for each study in Supplementary Table 1):

$$Y={\beta }_{0}+{\beta }_{G}G+{\beta }_{E}E+{\beta }_{G\times E}G\times E+{\beta }_{C}C$$

The 1df test was based on the null hypothesis H_0_: ${\beta }_{G\times E}=0$, while the 2df test was based on H_0_: ${{\beta }_{G}=\beta }_{G\times E}=0$. [92] To ensure robust estimates of covariance matrices and robust standard errors, studies of unrelated subjects used either the sandwich R package or ProbABEL genetic software [93]. Family studies used Mixed Model Analysis for Pedigrees and populations (MMAP), a comprehensive mixed model program that provides an optimized and flexible platform incorporating a wide range of covariance structures. Stage 2 studies carried out the same regressions, but only on the variants that reached suggestive significance (*P* < 10^−5^) in Stage 1 for any trait in population-specific or cross-population meta-analysis. For comparison, stage 1 studies also ran a main effect model (serum lipids as a function of the variant with adjustment for covariates and study-specific variables) and a main effect model additionally adjusted for the psychosocial factor.

### Population groups

Appropriate selection of population descriptors is a matter of considerable discussion in the field and consensus regarding optimal terms has not yet emerged. In this work, contributing studies were subdivided into population groups based on where individuals included in those studies were expected to cluster genetically to reduce the potential for spurious findings due to population structure and to maximize the potential for discovery within a population. Inclusion of samples within a particular cluster of genetic similarity was based on consultation with study teams given their expertise and understanding of the study population. Our approach includes African ancestry (AFR), Asian ancestry (ASN), Brazilian (BRZ), European ancestry (EUR), and admixed Hispanic/Latino and Native American participants (HISP). The AFR population group includes sub-Saharan Africans as well as participants with predominantly African ancestry living in the United States. The ASN population group includes participants of predominantly Asian ancestry living in East Asia, Singapore, or the United States. The EUR population group includes participants with predominantly European ancestry living in Europe or the United States. The HISP population group includes admixed Hispanic/Latino and Native American participants living in the United States. Brazilian individuals (BRZ) were analyzed separately after consultation with local researchers regarding the genetic clustering of these participants.

### Quality control and cross-population meta-analysis

We performed extensive study- and population-level quality control (QC) using the R package EasyQC for all GWAS results [94]. In study-level QC, allele frequencies for each study were compared visually to an ancestry-matched 1000 genomes reference panel to identify systematic errors in data preparation (no variants were excluded), and marker names were harmonized to ensure consistency across studies. Any resulting concerns were resolved in consultation with the contributing study. Variants were excluded if the imputation quality score was less than 0.5 or if 2×MAF×N_exposed_×imputation quality score was less than 20. Population-level QC was also conducted prior to meta-analysis to check for any outliers among included studies, which might suggest improper trait transformation or model specification, among other things.

We then conducted population-specific and cross-population meta-analysis in Stage 1 using the approach developed by Manning et al. [95] and implemented in METAL [95, 96]. This method performs a joint meta-analysis of the variant and the G×Psy exposure regression coefficients and then uses a 2df test to identify genetic variants driven jointly by main and interaction effects. Additionally, we used the inverse-variance weighted meta-analysis implemented in METAL to meta-analyze G×Psy interaction coefficients alone using a 1df test. Variants in the Stage 1 meta-analysis had to be present in at least 2 cohorts or at least 3000 individuals for AFR and EUR, with a lower threshold (*n* = 2000) set for ASN, BRZ, and HISP because of the smaller number of individuals available in these ancestries. In Stage 2, we used the same approach as in Stage 1 to perform population-specific and cross-population meta-analyses. After combining results from Stages 1 and 2, variants with *P* < 5.0 × 10^−8^ for either the 2df joint test of the main effect and the interaction or the 1df test of the interaction were considered significant. Results with a heterozygosity p-value < 0.05 were evaluated further and excluded if results were driven by a single cohort.

The novelty of associated loci was determined by comparison to the recent Global Lipids Genetic Consortium results for GWAS meta-analyses including approximately 1.65 million individuals with notable inclusion of those of diverse ancestral backgrounds [1]. The 95% credible sets from the meta-analyses of all lipids traits (available at http://csg.sph.umich.edu/willer/public/glgc-lipids2021/results/credible_sets/) were compiled. Variants were considered novel if they were 500 kb from all variants listed in this list, as well as those reported in other major publications [34–39].

### Identification of independent genomic loci and functional annotation

Identification of genetic loci related to each of the three serum lipids and functional annotation was accomplished using Functional Mapping and Annotation of GWAS (FUMA) v1.5.6 (http://fuma.ctglab.nl/) [97]. Variants were grouped into genomic loci using an R^2^ < 0.6 (1000 Genomes, Phase 3 ALL as the reference population) and a merge distance of 250 kb. Functional annotation was conducted using output from the set of tools incorporated within FUMA, including RegulomeDB score, Combined Annotation Dependent Deletion (CADD) score [98], 15-core chromatin state (ChromHMM) [41, 99, 100], and expression Quantitative Trait Loci (eQTL) on the variants from lead associations as well as those in LD with those variants (R^2^ > 0.1), using all tissues and all included databases (including GTex, BloodeQTL, BIOS, and BRAINEAC).

### Druggability analysis

We first used the Drug-Gene Interaction database (DGIdb; v4.2.0) to query psychosocial factors-lipid interacting genes to determine the potential druggability of the candidate gene targets. We annotated genes for implicated pathways and functions using the Kyoto Encyclopedia of Genes and Genomes (KEGG) database. We annotated the druggability target categories and queried all interacting drugs reported in 41 databases (BaderLabGenes, CarisMolecularIntelligence, dGene, FoundationOneGenes, GO, HingoraniCasas, HopkinsGroom, HumanProteinAtlas, IDG, MskImpact, Oncomine, Pharos, RussLampel, Tempus, CGI, CIViC, COSMIC, CancerCommons, ChEMBL, ChemblDrugs, ChemblInteractions, ClearityFoundationBiomarkers, ClearityFoundationClinicalTrial, DTC, DoCM, DrugBank, Ensembl, Entrez, FDA, GuideToPharmacology, JAX-CKB, MyCancerGenome, MyCancerGenomeClinicalTrial, NCI, OncoKB, PharmGKB, TALC, TEND, TTD, TdgClinicalTrial, Wikidata). We queried protein targets for available active ligands in ChEMBL. We queried gene targets in the druggable genome using the most recent druggable genome list established by the NIH Illuminating the Druggable Genome Project (https://github.com/druggablegenome/IDGTargets) available through the Pharos web platform. We also queried FDA-approved drugs, late-stage clinical trials, and disease indications in the DrugBank, ChEMBL, ClinicalTrials.gov databases and provided results for the top MESH and DrugBank indications and clinical trials.

## Supplementary information

Supplementary Materials

Supplemental Tables

## Data Availability

All summary results are available in the GWAS Catalog with the following Accession IDs: AFR.HDLC.ANXT.2df (GCST90570645); AFR.HDLC.ANXT.1df (GCST90570646); AFR.HDLC.DEPR.2df (GCST90570647); AFR.HDLC.DEPR.1df (GCST90570648); AFR.HDLC.SOCS.2df (GCST90570649); AFR.HDLC.SOCS.1df (GCST90570650); AFR.LDLC.ANXT.2df (GCST90570651); AFR.LDLC.ANXT.1df (GCST90570652); AFR.LDLC.DEPR.2df (GCST90570653); AFR.LDLC.DEPR.1df (GCST90570654); AFR.TG.ANXT.2df (GCST90570655); AFR.TG.ANXT.1df (GCST90570656); AFR.TG.DEPR.2df (GCST90570657); AFR.TG.DEPR.1df (GCST90570658); ASN.HDLC.DEPR.2df (GCST90570659); ASN.HDLC.DEPR.1df (GCST90570660); ASN.LDLC.DEPR.2df (GCST90570661); ASN.LDLC.DEPR.1df (GCST90570662); ASN.TG.DEPR.2df (GCST90570663); ASN.TG.DEPR.1df (GCST90570664); EUR.HDLC.ANXT.2df (GCST90570665); EUR.HDLC.ANXT.1df (GCST90570666); EUR.HDLC.DEPR.2df (GCST90570667); EUR.HDLC.DEPR.1df (GCST90570668); EUR.HDLC.SOCS.2df (GCST90570669); EUR.HDLC.SOCS.1df (GCST90570670); EUR.LDLC.ANXT.2df (GCST90570671); EUR.LDLC.ANXT.1df (GCST90570672); EUR.LDLC.DEPR.2df (GCST90570673); EUR.LDLC.DEPR.1df (GCST90570674); EUR.LDLC.SOCS.2df (GCST90570675); EUR.LDLC.SOCS.1df (GCST90570676); EUR.TG.ANXT.2df (GCST90570677); EUR.TG.ANXT.1df (GCST90570678); EUR.TG.DEPR.2df (GCST90570679); EUR.TG.DEPR.1df (GCST90570680); EUR.TG.SOCS.2df (GCST90570681); EUR.TG.SOCS.1df (GCST90570682); HISP.HDLC.DEPR.2df (GCST90570683); HISP.HDLC.DEPR.1df (GCST90570684); HISP.HDLC.SOCS.2df (GCST90570685); HISP.HDLC.SOCS.1df (GCST90570686); HISP.LDLC.DEPR.2df (GCST90570687); HISP.LDLC.DEPR.1df (GCST90570688); HISP.LDLC.SOCS.2df (GCST90570689); HISP.LDLC.SOCS.1df (GCST90570690); HISP.TG.DEPR.2df (GCST90570691); HISP.TG.DEPR.1df (GCST90570692); HISP.TG.SOCS.2df (GCST90570693); HISP.TG.SOCS.1df (GCST90570694); CPMA.HDLC.ANXT.2df (GCST90570695); CPMA.HDLC.ANXT.1df (GCST90570696); CPMA.HDLC.DEPR.2df (GCST90570697); CPMA.HDLC.DEPR.1df (GCST90570698); CPMA.HDLC.SOCS.2df (GCST90570699); CPMA.HDLC.SOCS.1df (GCST90570700); CPMA.LDLC.ANXT.2df (GCST90570701); CPMA.LDLC.ANXT.1df (GCST90570702); CPMA.LDLC.DEPR.2df (GCST90570703); CPMA.LDLC.DEPR.1df (GCST90570704); CPMA.LDLC.SOCS.2df (GCST90570705); CPMA.LDLC.SOCS.1df (GCST90570706); CPMA.TG.ANXT.2df (GCST90570707); CPMA.TG.ANXT.1df (GCST90570708); CPMA.TG.DEPR.2df (GCST90570709); CPMA.TG.DEPR.1df (GCST90570710); CPMA.TG.SOCS.2df (GCST90570711); CPMA.TG.SOCS.1df (GCST90570712)
