# Supplementary material for: Multi-ancestry genome-wide association analyses incorporating SNP-by-psychosocial interactions identify novel loci for serum lipids
Source: Transl Psychiatry. 2025 Jun 20;15:207. doi: 10.1038/s41398-025-03418-z (PMC12179276; doi:10.1038/s41398-025-03418-z)
Supplement: Supplementary file 1 — Supplementary Materials [file 41398_2025_3418_MOESM1_ESM.pdf]

Supplementary Material

Multi-Ancestry Genome-Wide Association Analyses Incorporating SNP-by-Psychosocial Interactions Identify Novel Loci for Serum Lipids

Amy R. Bentley, Michael R. Brown, Solomon K. Musani, Karen L. Schwander, Thomas W. Winkler, *et al.*

Contents

Supplementary Tables *Excel File*

Supplementary Figures

- SF1: Manhattan Plots p. 2
- SF2: QQ Plots p. 15
- SF3: Forest Plot of Statistically Significant 1df Interaction Results p. 20
- SF4: Forest Plot of Statistically Significant 2df Main Effect and Interaction Results p. 23

Supplementary Note

- Study Descriptions p. 29
- Study Acknowledgments p. 42

Supplemental Figures

Supplemental Figure 1: Manhattan Plots. Shown are Manhattan plots for all meta-analyses conducted for both the 1df test of interaction (orange/gray) and the 2df test of main effect and interaction (blue/gray). Stage 1 + 2 results are given where available, with Stage 1 only results included for all variants for which follow-up in Stage 2 was not sought. Lead novel associations from either the 1df test of interaction (**Table 1**) or the 2df test of main effect and interaction (**Table 2**) are annotated with the name of the nearest gene.

AFR

HDLC

1df Test of Interaction | **2df Joint Test of Main Effect and Interaction**

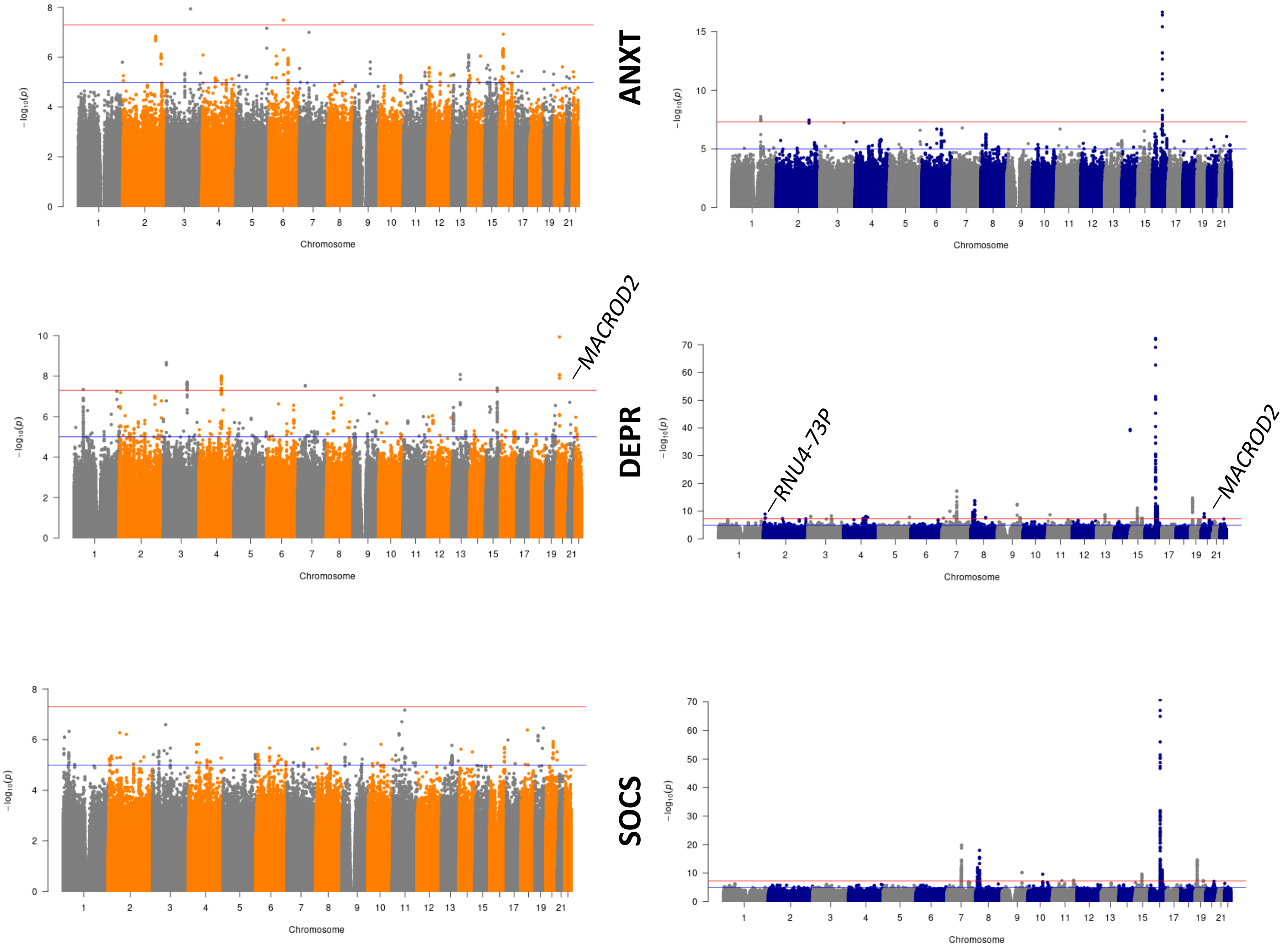

AFR

LDLC

1df Test of Interaction

2df Joint Test of Main Effect and Interaction

ANXT

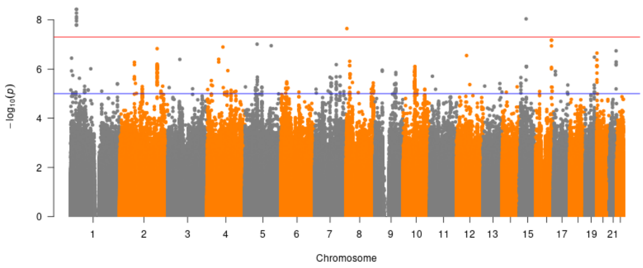

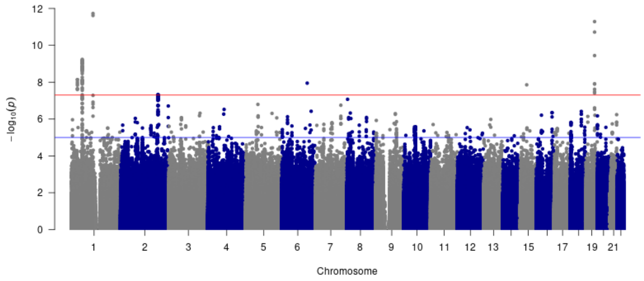

DEPR

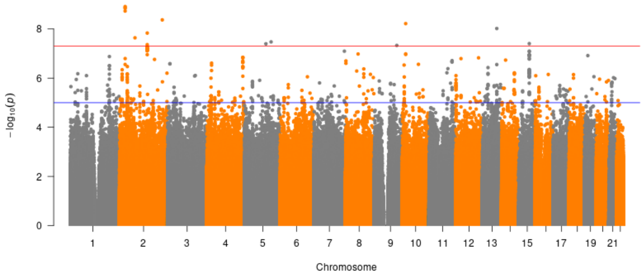

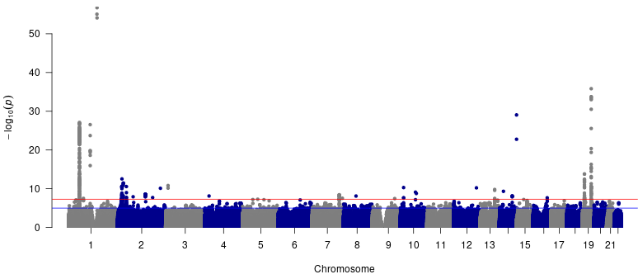

SOCS

None

None

AFR

TG

1df Test of Interaction

2df Joint Test of Main Effect and Interaction

ANXT

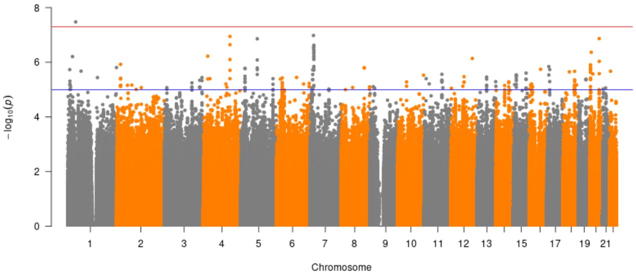

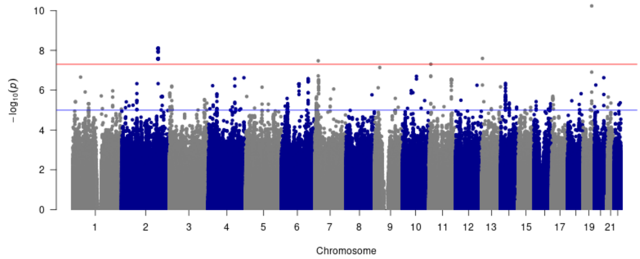

DEPR

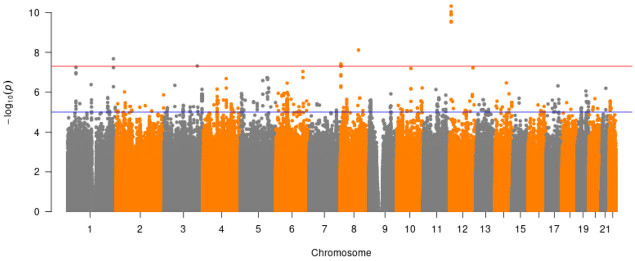

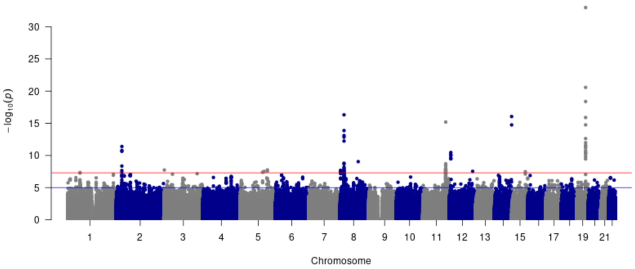

SOCS

None

None

ASN

Note: ASN cohorts only had DEPR results.

1df Test of Interaction

2df Joint Test of Main Effect and Interaction

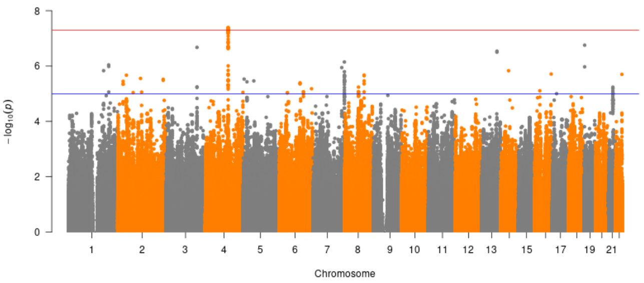

HDLC-DEPR

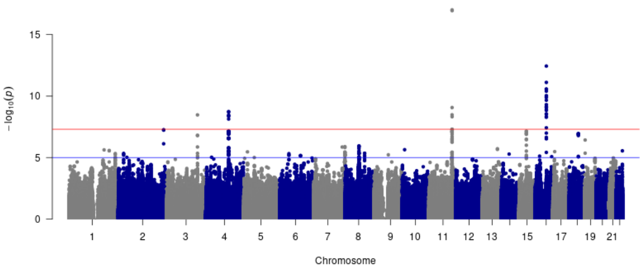

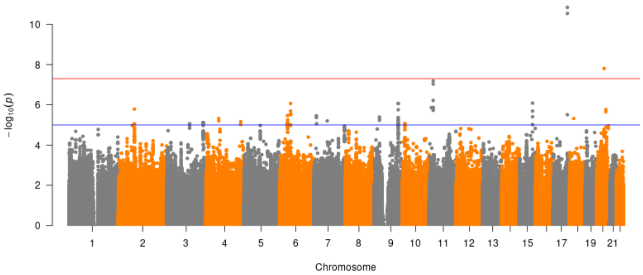

LDLC-DEPR

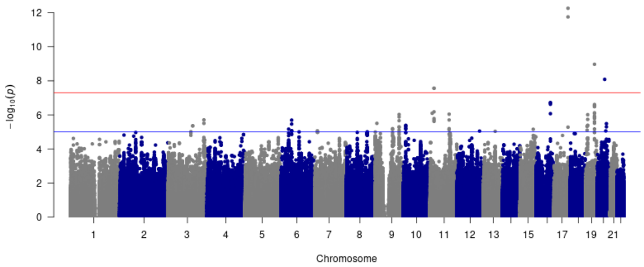

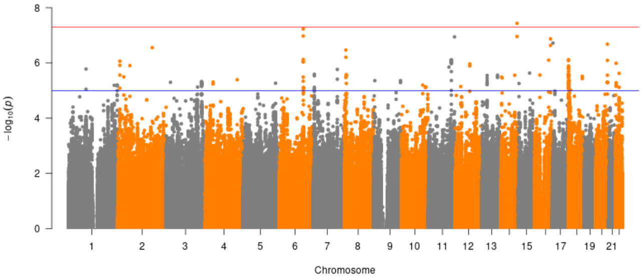

TG-DEPR

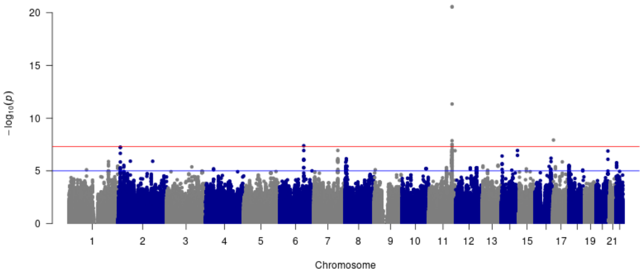

EUR

HDLC

1df Test of Interaction

2df Joint Test of Main Effect and Interaction

ANXT

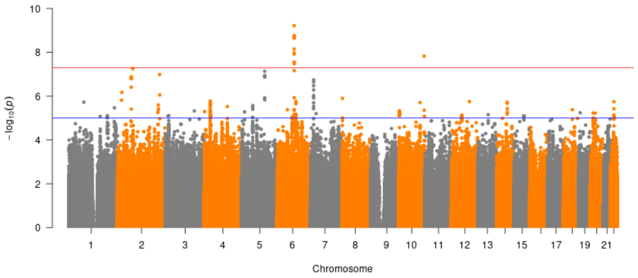

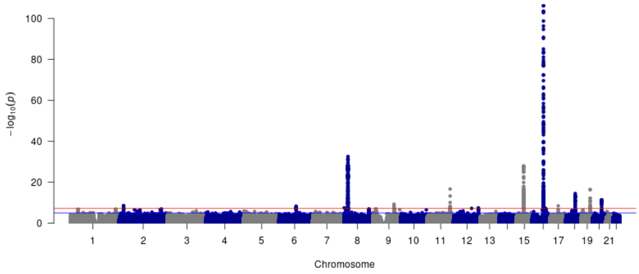

DEPR

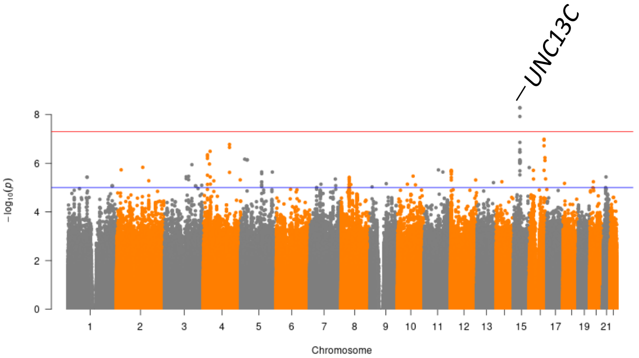

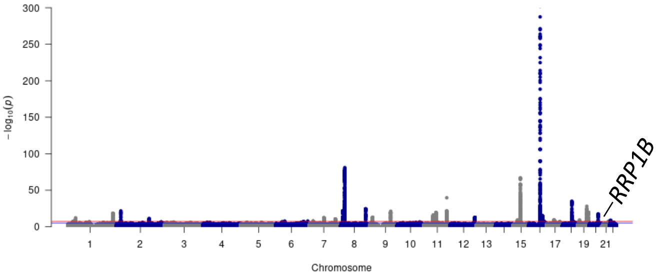

SOCS

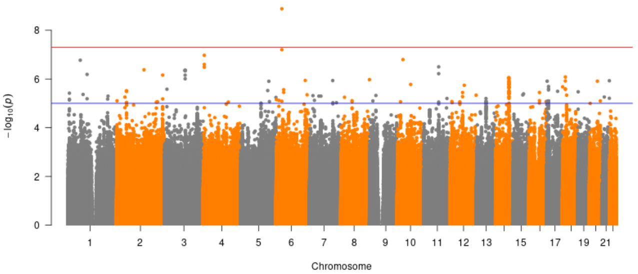

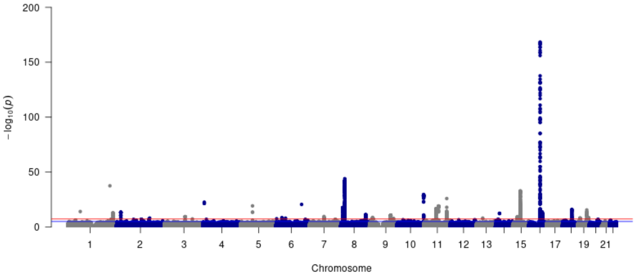

EUR

LDLC

1df Test of Interaction

2df Joint Test of Main Effect and Interaction

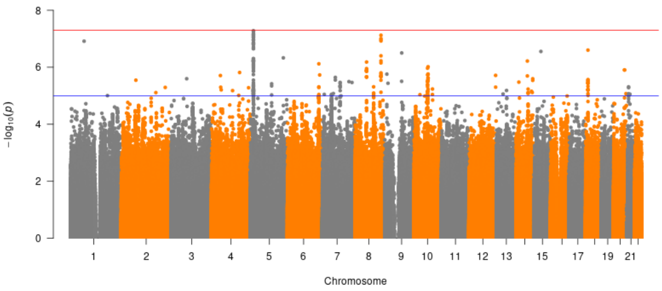

ANXT

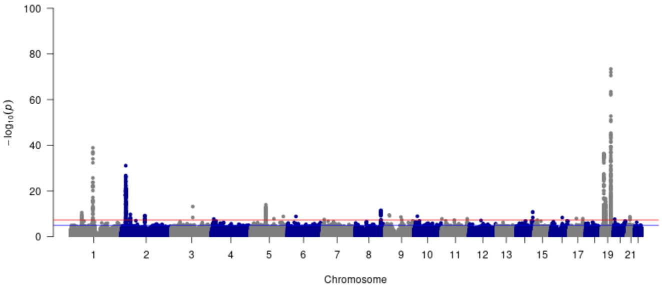

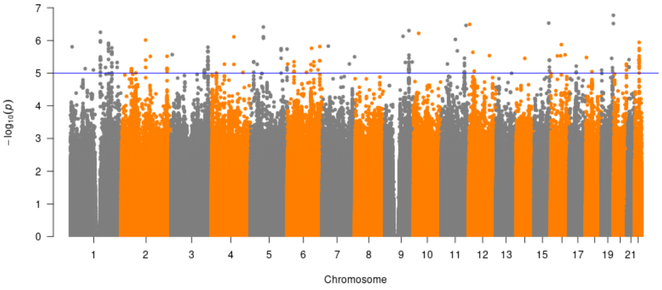

DEPR

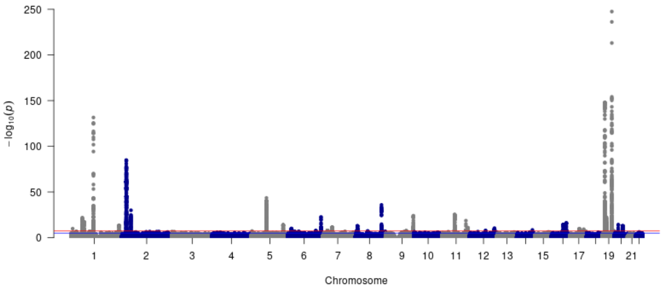

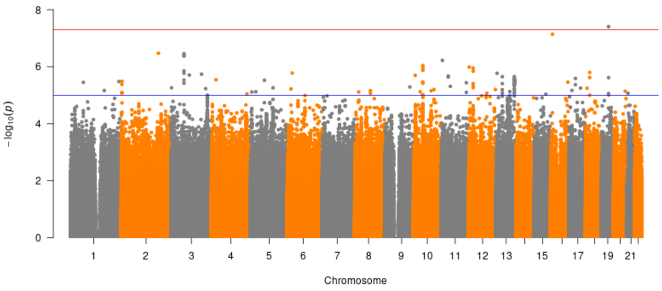

SOCS

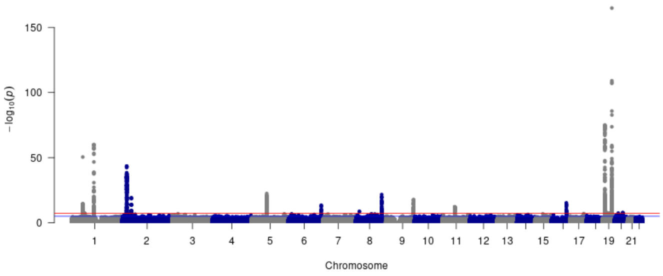

EUR

TG

1df Test of Interaction

2df Joint Test of Main Effect and Interaction

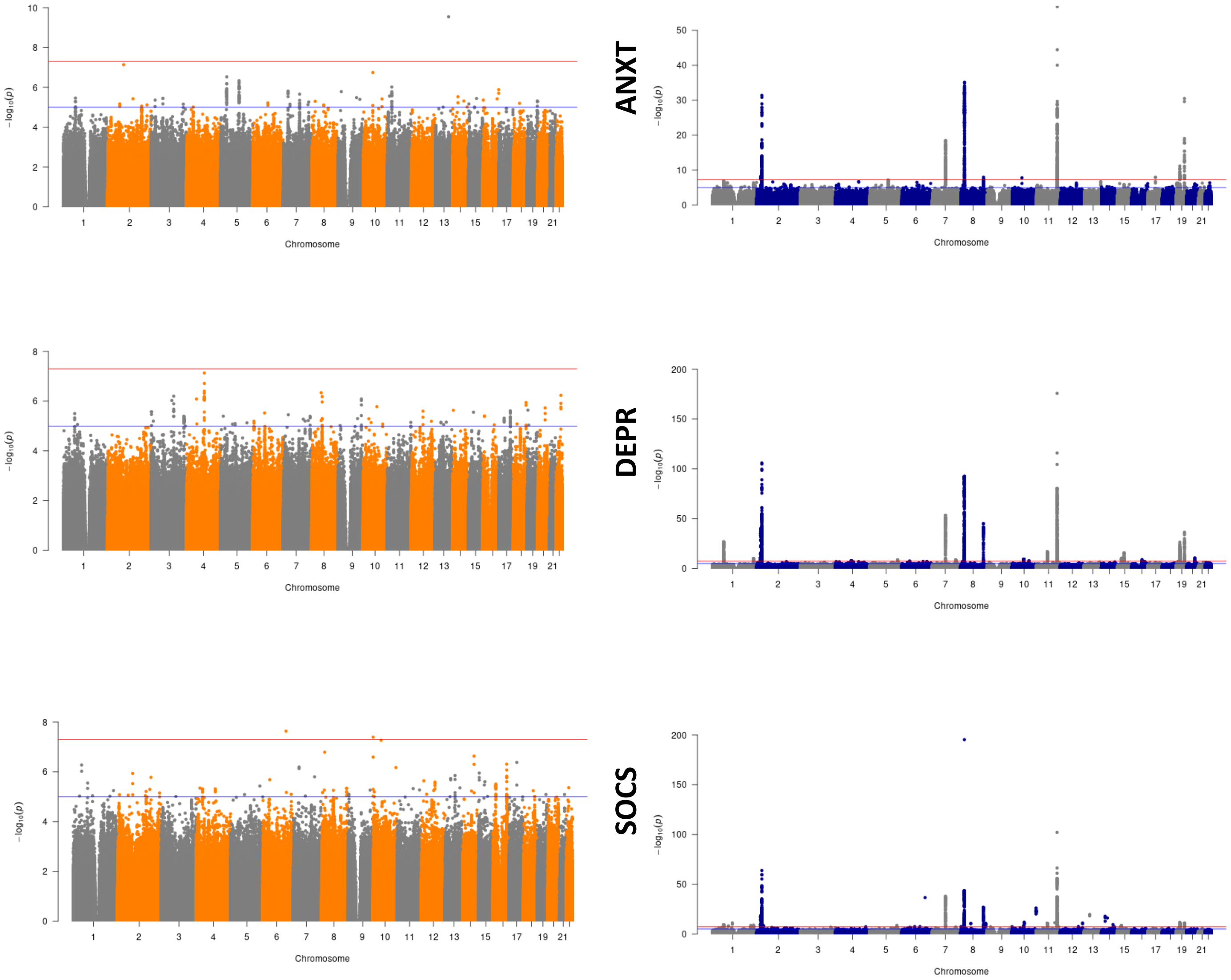

HISP

HDLC

1df Test of Interaction

2df Joint Test of Main Effect and Interaction

ANXT

None

None

DEPR

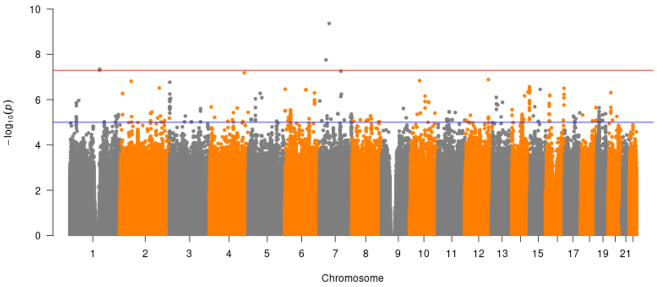

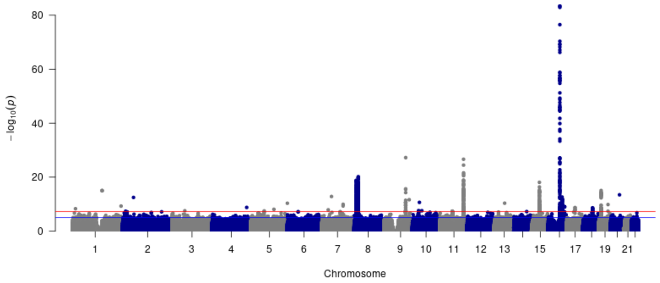

SOCS

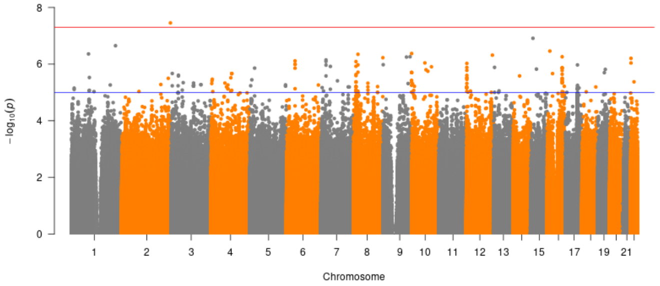

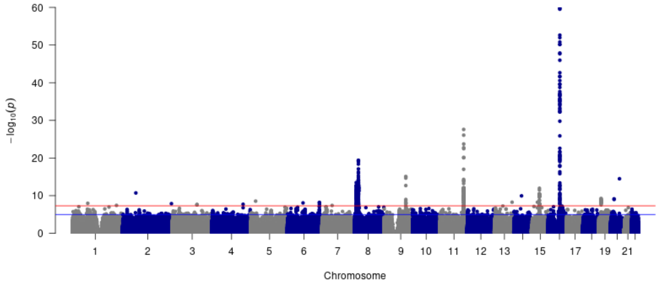

HISP

LDLC

1df Test of Interaction

2df Joint Test of Main Effect and Interaction

ANXT

None

None

DEPR

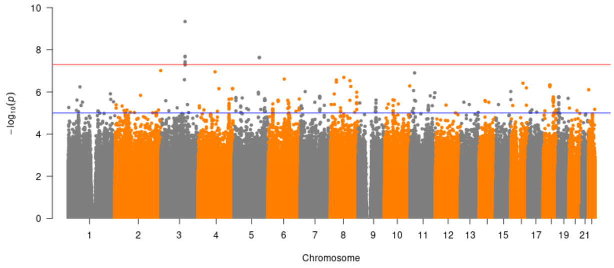

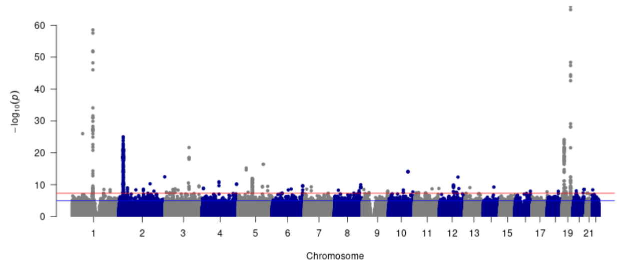

SOCS

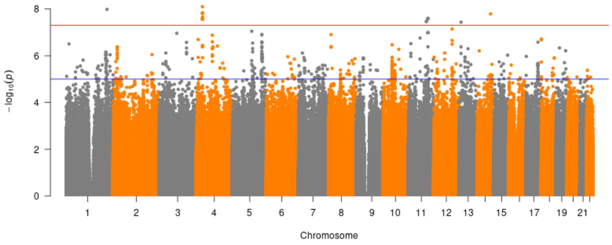

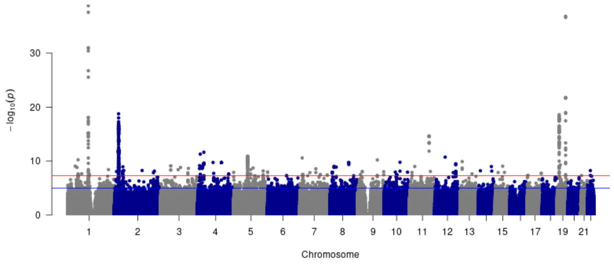

HISP

TG

1df Test of Interaction

2df Joint Test of Main Effect and Interaction

ANXT

None

None

DEPR

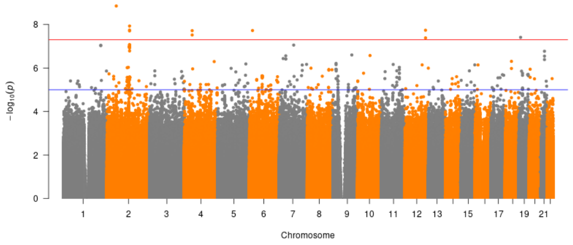

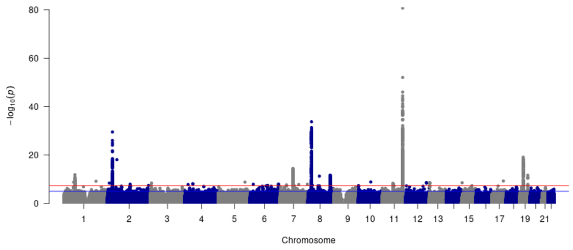

SOCS

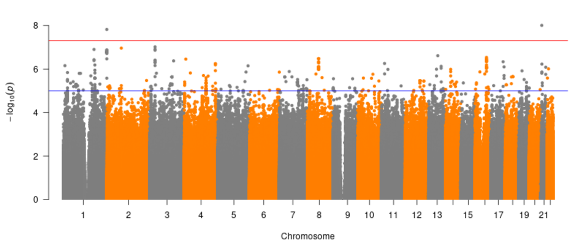

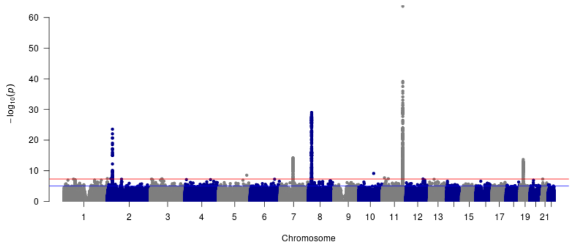

CPMA

HDLC

1df Test of Interaction

2df Joint Test of Main Effect and Interaction

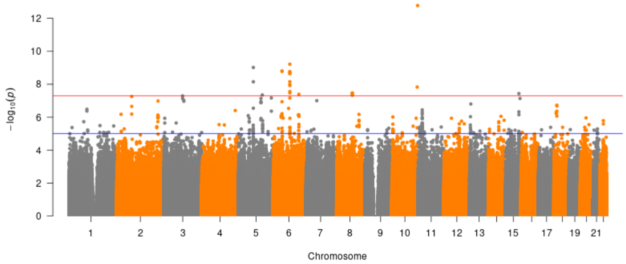

ANXT

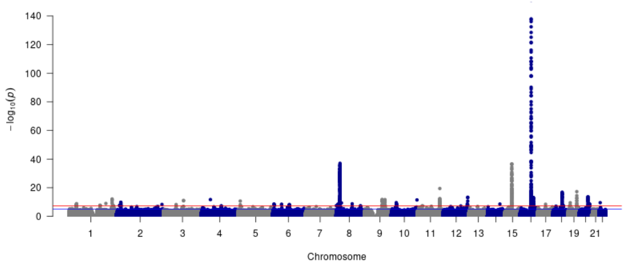

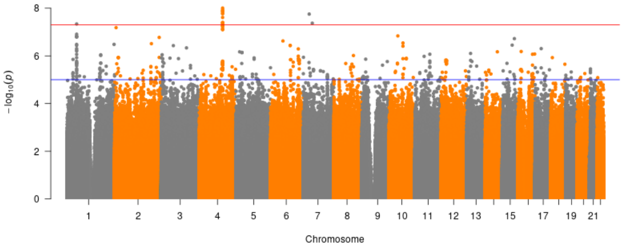

DEPR

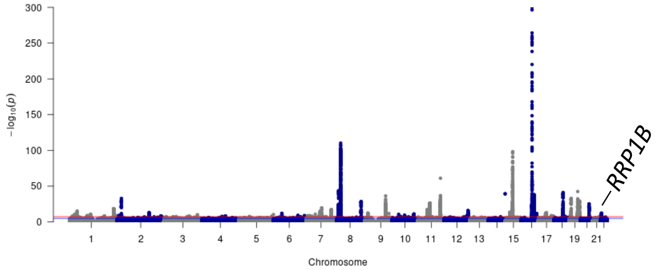

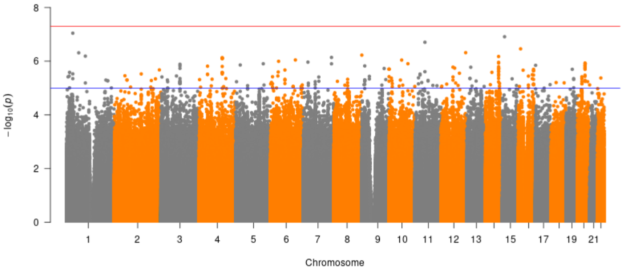

SOCS

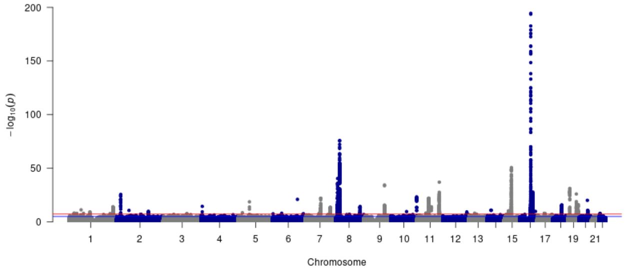

CPMA

LDLC

1df Test of Interaction

2df Joint Test of Main Effect and Interaction

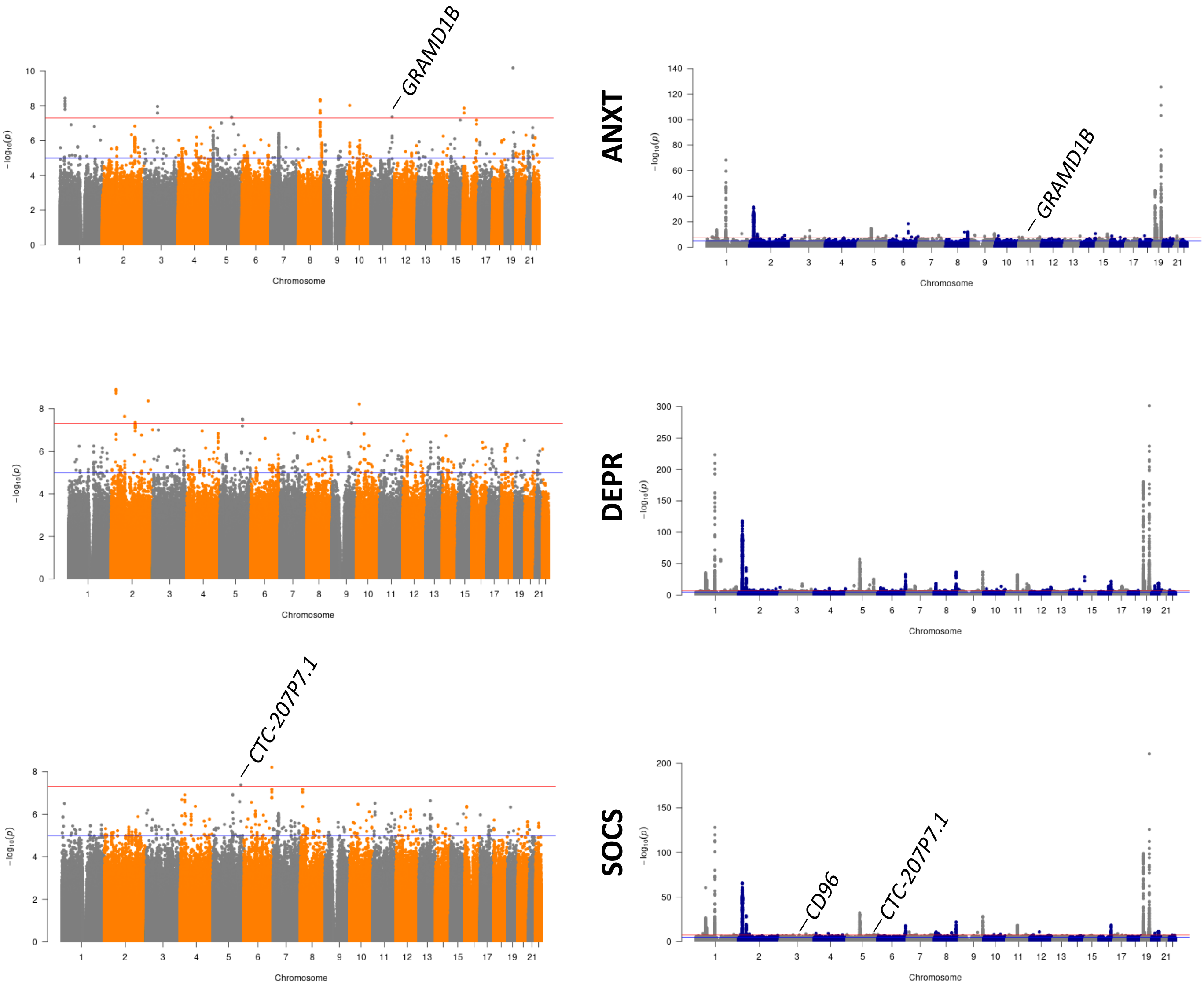

CPMA

TG

1df Test of Interaction

2df Joint Test of Main Effect and Interaction

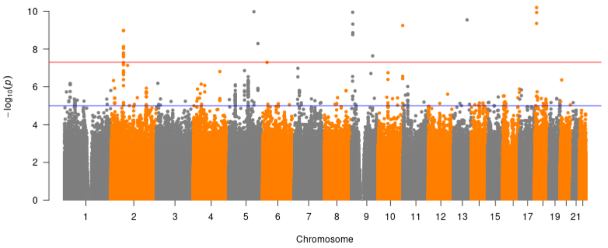

ANXT

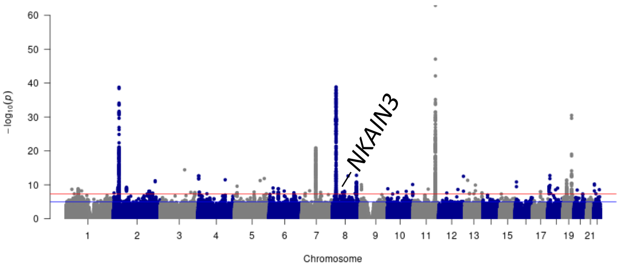

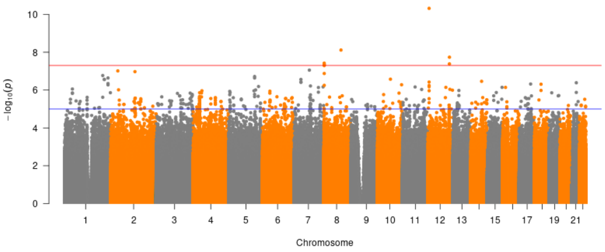

DEPR

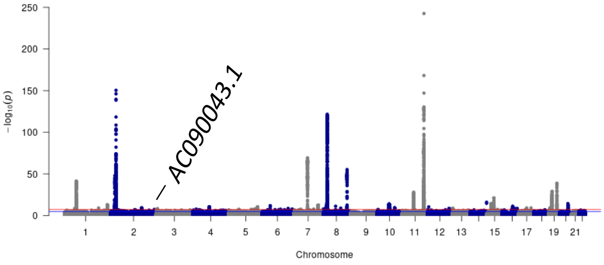

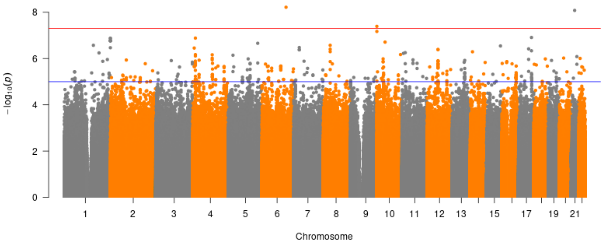

SOCS

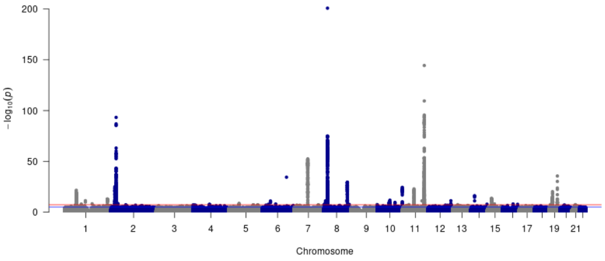

Supplemental Figure 2: QQ Plots. Shown are QQ plots for all meta-analyses conducted for both the 1df test of interaction (orange) and the 2df test of main effect and interaction (blue). Stage 1 + 2 results are given where available, with Stage 1 only results included for all variants for which follow-up in Stage 2 was not sought.

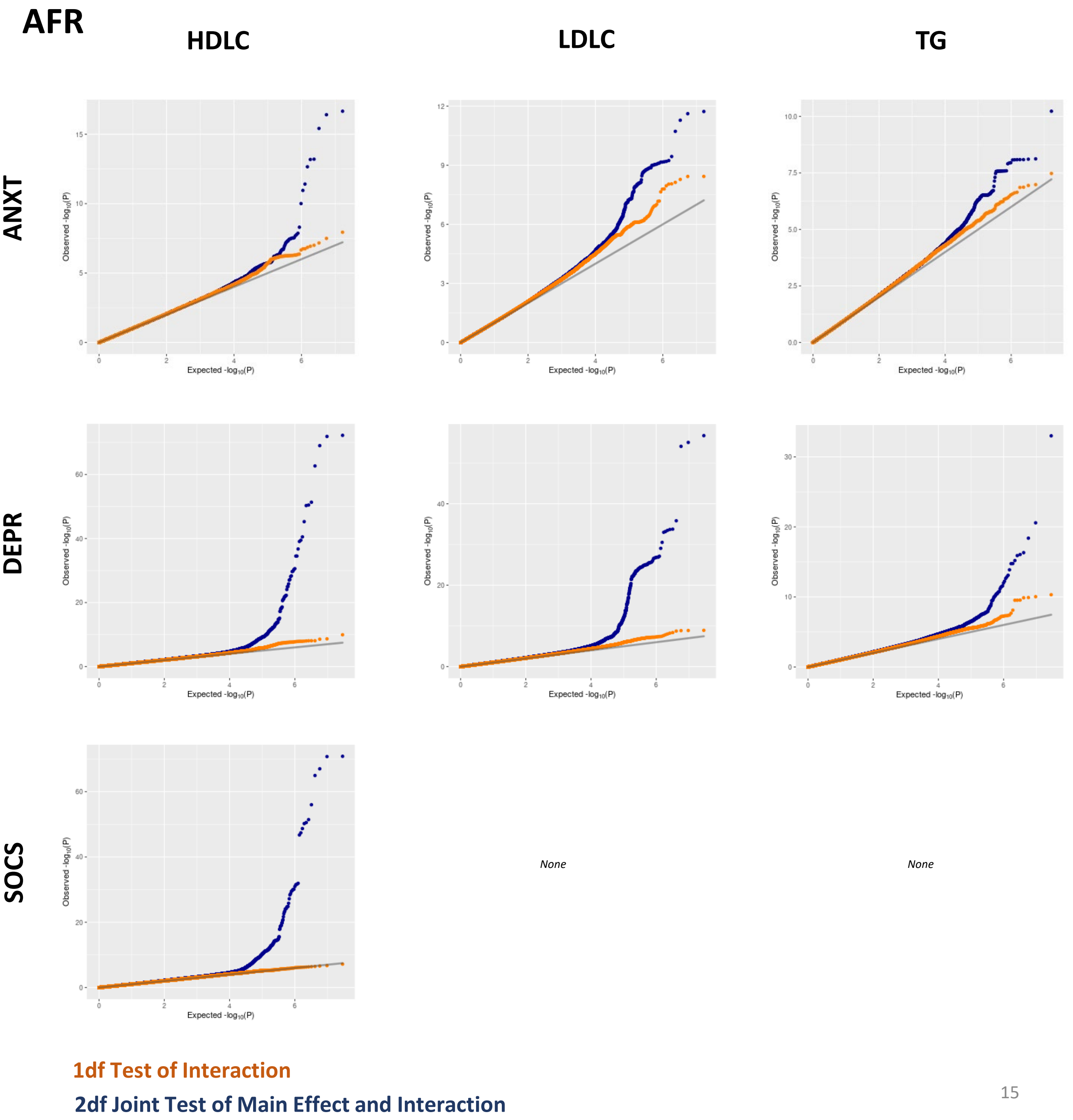

ASN

	HDLC	LDLC	TG
ANXT	*None*	*None*	*None*
DEPR	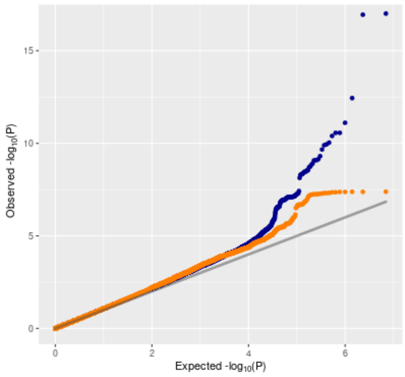	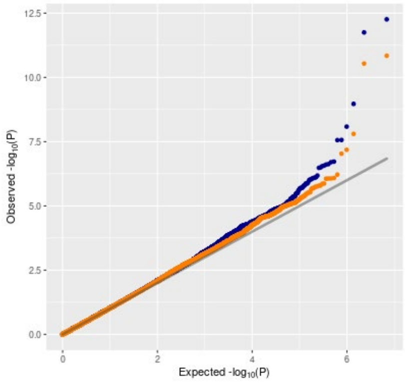	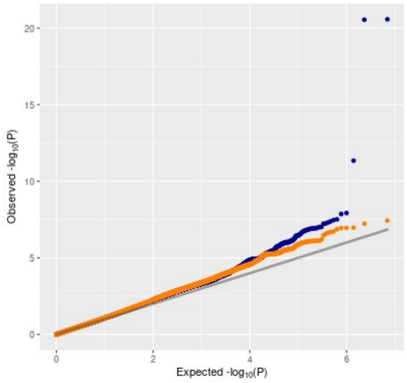
SOCS	*None*	*None*	*None*

1df Test of Interaction

2df Joint Test of Main Effect and Interaction

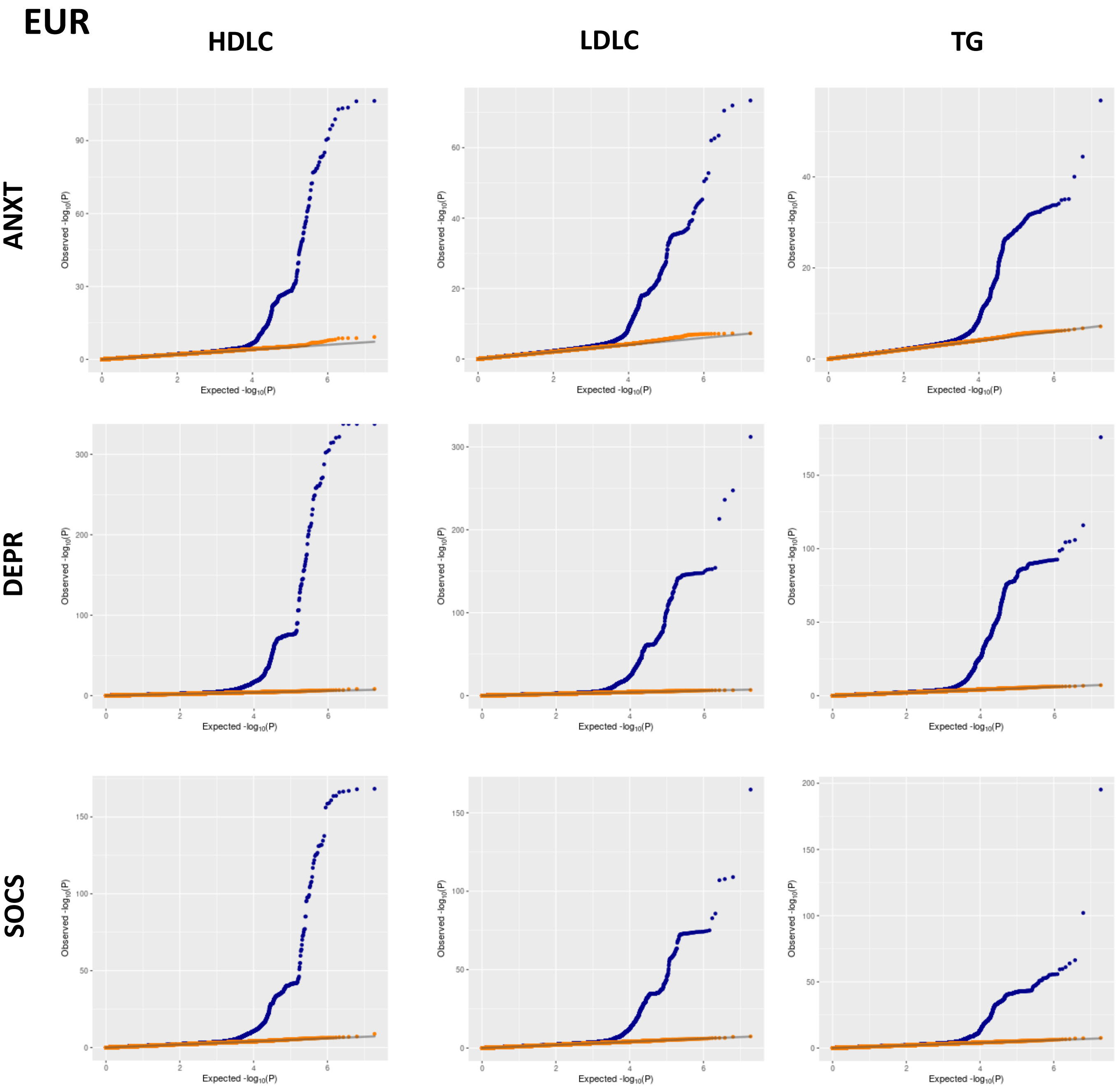

1df Test of Interaction

2df Joint Test of Main Effect and Interaction

HISP

HDLC | **LDLC** | **TG**

ANXT

None | *None* | *None*

DEPR

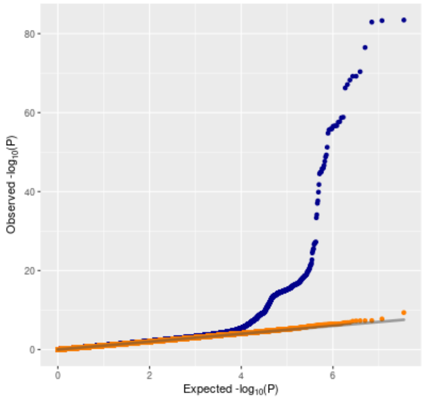

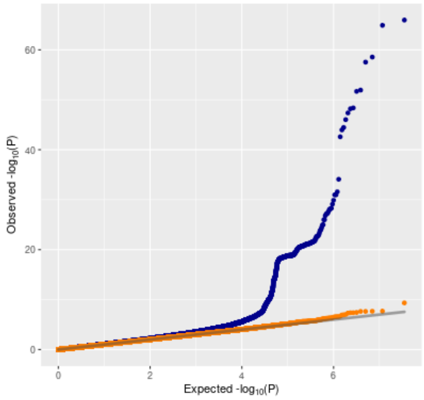

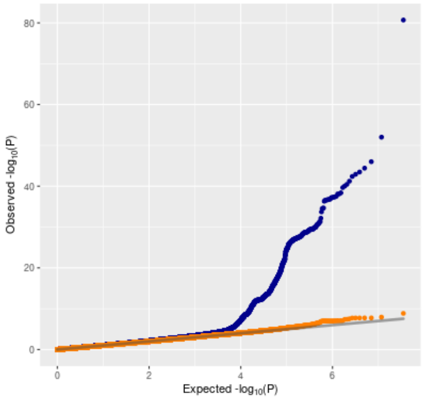

SOCS

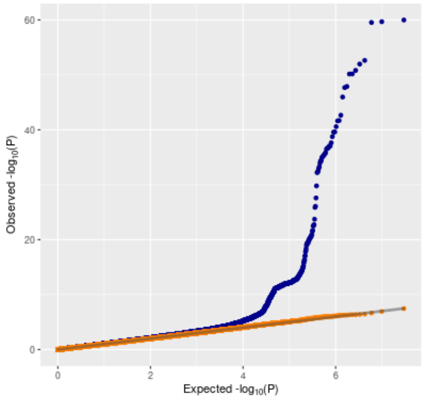

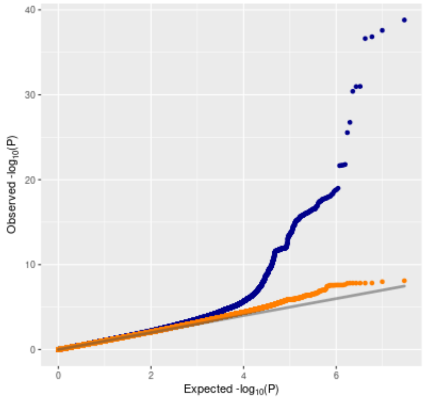

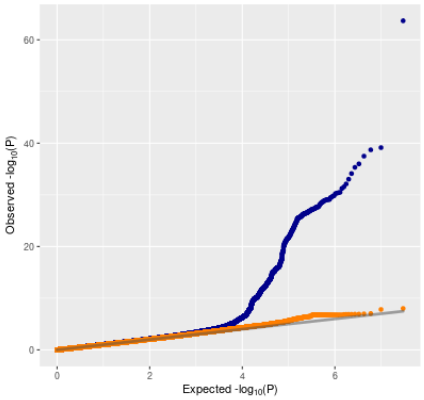

1df Test of Interaction

2df Joint Test of Main Effect and Interaction

CPMA

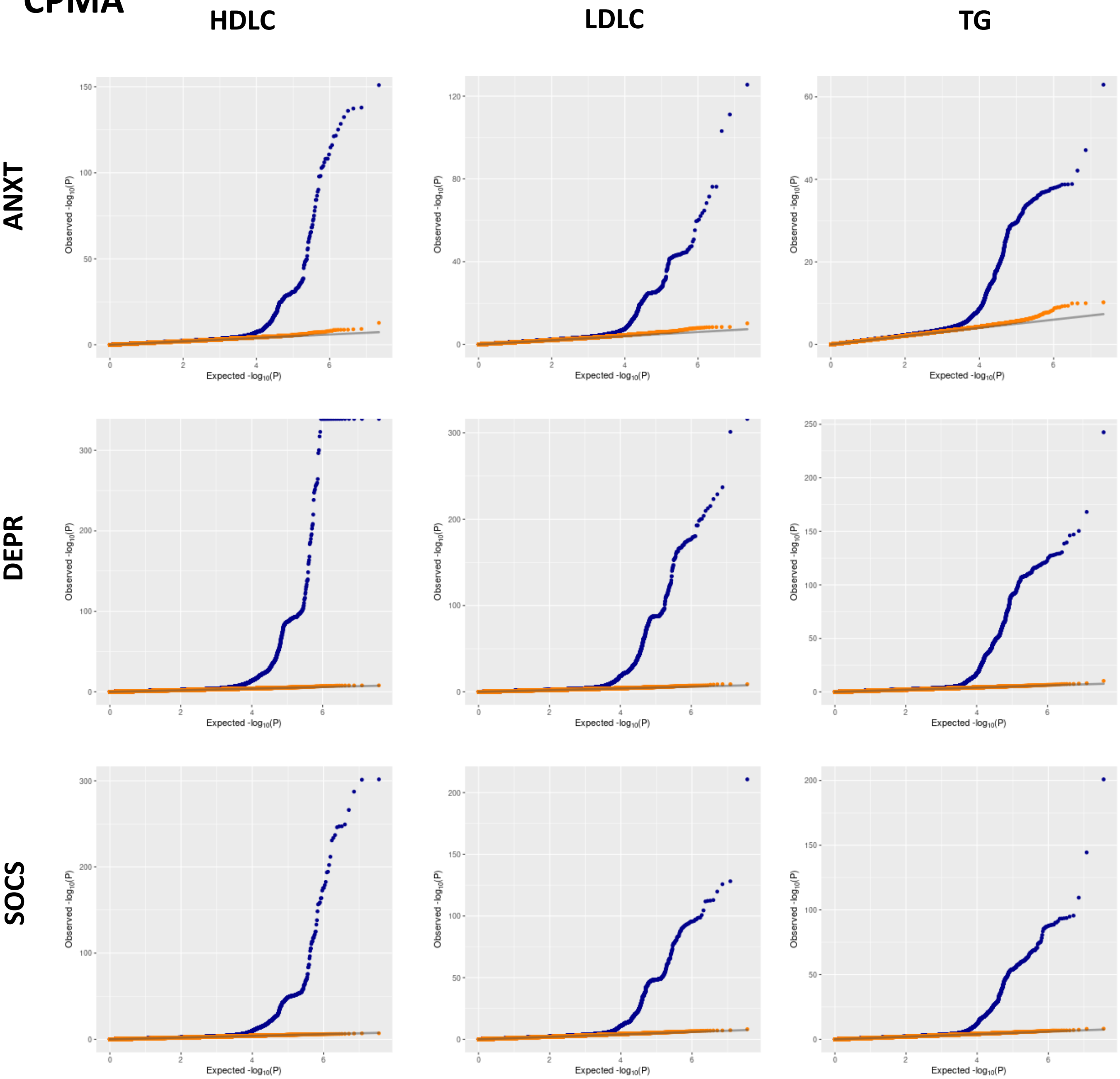

1df Test of Interaction

2df Joint Test of Main Effect and Interaction

Supplemental Figure 3: Forest Plot of Statistically Significant 1df Interaction Results. Shown are all individual study results with data for the given locus (when the number of contributing studies is large, only meta-analysis results are shown for readability). If only a population-specific result is shown, then that locus was not present or did not pass filters in contributing studies in any other population. Shown in blue is the beta-coefficient for the multiplicative interaction term between the variant and the listed psychosocial factor on lipid concentration. Box size represents the precision of the estimate, with larger boxes shown for results with lower variance.

A.

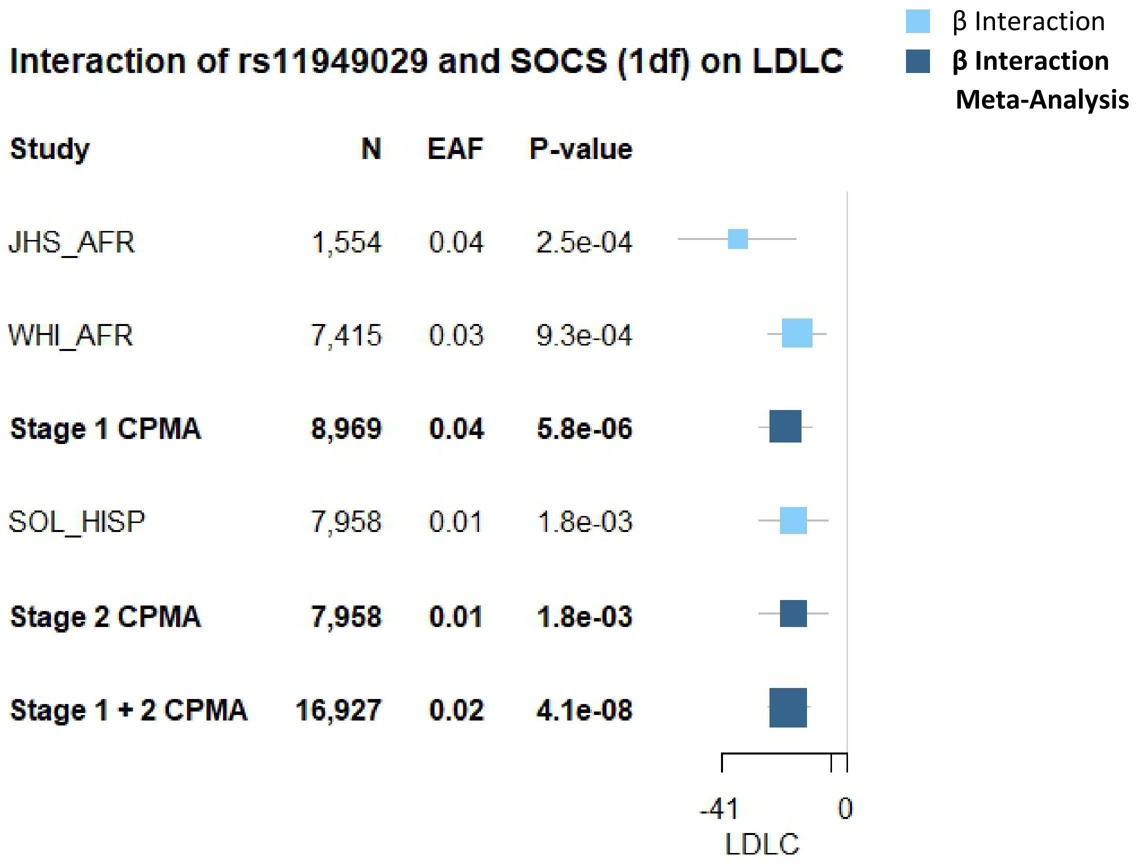

B.

Interaction of rs59808825 and ANXT (1df) on LDLC

β Interaction
β Interaction Meta-Analysis

Study	N	EAF	P-value
BAEPENDI_BRZ	743	0.04	6.8e-07
PELOTAS_BRZ	2,693	0.04	3.2e-03
**Stage 1 CPMA**	**3,436**	**0.04**	**1.7e-07**
NESDA_EUR	2,537	0.02	0.07
**Stage 2 CPMA**	**2,537**	**0.02**	**0.07**
**Stage 1 + 2 CPMA**	**5,973**	**0.03**	**4.3e-08**

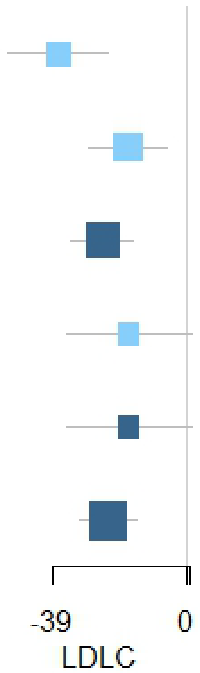

C.

Interaction of rs61248562 and DEPR (1df) on HDLC

β Interaction
β Interaction Meta-Analysis

Study	N	EAF	P-value
FHS_EUR	6,788	0.02	6.5e-08
NEO_EUR	5,717	0.01	0.01
**Stage 1 EUR**	**12,505**	**0.02**	**7.6e-09**
NESDA_EUR	2,547	0.01	0.25
**Stage 2 EUR**	**2,547**	**0.01**	**0.25**
**Stage 1 + 2 EUR**	**15,052**	**0.02**	**5.2e-09**
**Stage 1 + 2 AFR**	**13,069**	**0.16**	**0.30**
**Stage 1 + 2 HISP**	**15,977**	**0.07**	**0.33**
**Stage 1 + 2 CPMA**	**46,812**	**0.1**	**1.5e-03**

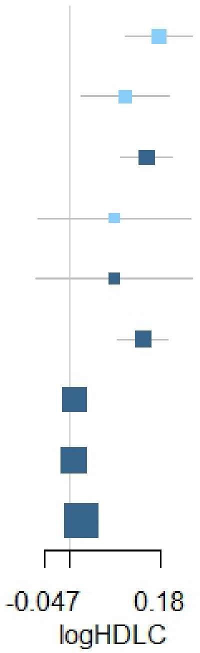

D.

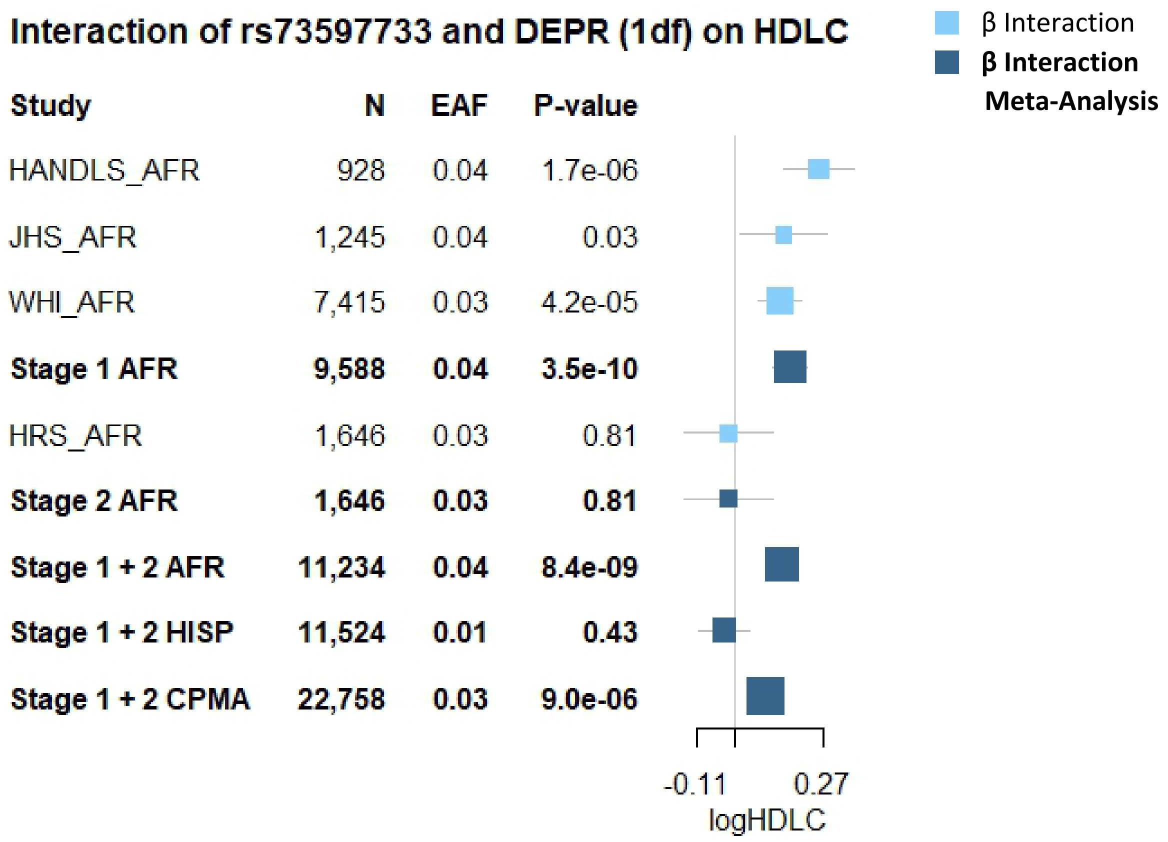
Interaction of rs73597733 and DEPR (1df) on HDLC
β Interaction
β Interaction Meta-Analysis
Study N EAF P-value
HANDLS_AFR 928 0.04 1.7e-06
JHS_AFR 1,245 0.04 0.03
WHI_AFR 7,415 0.03 4.2e-05
Stage 1 AFR 9,588 0.04 3.5e-10
HRS_AFR 1,646 0.03 0.81
Stage 2 AFR 1,646 0.03 0.81
Stage 1 + 2 AFR 11,234 0.04 8.4e-09
Stage 1 + 2 HISP 11,524 0.01 0.43
Stage 1 + 2 CPMA 22,758 0.03 9.0e-06
-0.11
0.27
logHDLC

Supplemental Figure 4: Forest Plot of Statistically Significant 2df Main Effect and Interaction Results. Shown are all individual study results with data for the given locus (when the number of contributing studies is large, only meta-analysis results are shown for readability). If only a population-specific result is shown, then that locus was not present or did not pass filters in contributing studies in any other population. Shown in blue is the beta-coefficient for the multiplicative interaction term between the variant and the listed psychosocial factor on lipid concentration, while the orange is the beta-coefficient for the main effect term in the same model. Box size represents the precision of the estimate, with larger boxes shown for results with lower variance.

A.

Interaction of rs6730082 and DEPR (2df) on HDLC

Study	N	EAF	P-value
ARIC_AFR	314	0.25	0.35
CARDIA_AFR	796	0.26	0.04
CHS_AFR	726	0.24	0.86
HABC_AFR	1,094	0.25	0.38
HANDLS_AFR	928	0.26	0.03
JHS_AFR	1,245	0.27	0.50
MESA_AFR	1,645	0.26	0.60
WHI_AFR	7,415	0.25	3.0e-04
**Stage 1 AFR**	**14,163**	**0.25**	**1.2e-05**
GENESTAR_AFR	1,077	0.27	9.9e-04
HRS_AFR	1,646	0.28	0.04
**Stage 2 AFR**	**2,723**	**0.27**	**7.0e-04**
**Stage 1 + 2 AFR**	**16,886**	**0.26**	**2.5e-08**
PELOTAS_BRZ	2,714	0.03	0.97
WHI_HISP	2,998	0.03	0.59
**Stage 1 + 2 CPMA**	**22,598**	**0.2**	**2.3e-07**

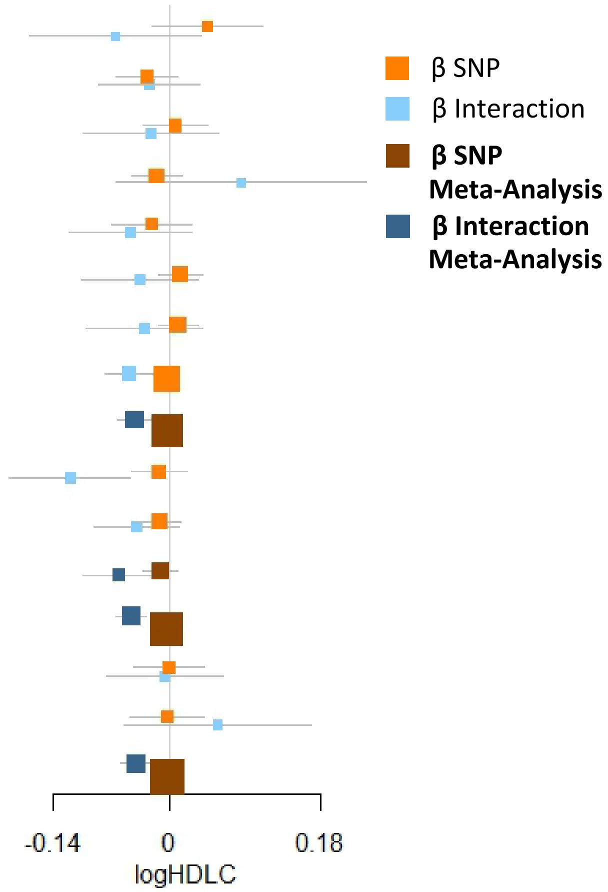

B.

Interaction of rs142378953 and DEPR (2df) on TG

Study	N	EAF	P-value
WHI_AFR	7,415	0.01	1.8e-08
**Stage 1 CPMA**	**7,415**	**0.01**	**1.8e-08**
SOL_HISP	11,496	0.002	0.26
**Stage 2 CPMA**	**11,496**	**0.002**	**0.26**
**Stage 1+2 CPMA**	**18,911**	**0.01**	**4.9e-08**

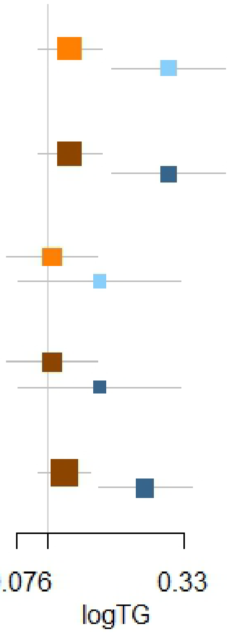

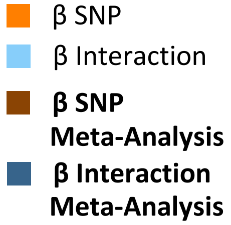

C.

Interaction of rs34636484 and SOCS (2df) on LDLC

Study	N	EAF	P-value
ARIC_EUR	7,366	0.04	5.7e-05
CARDIA_EUR	1,617	0.04	0.46
CHS_EUR	2,939	0.03	0.77
MESA_EUR	2,541	0.04	0.03
WHI-GARNET_EUR	3,635	0.03	5.7e-03
WHI-WHIMS_EUR	4,955	0.03	3.1e-03
WHI_HISP	2,998	0.02	0.45
**Stage 1 CPMA**	**26,051**	**0.03**	**2.5e-08**
KORA_F3_EUR	2,639	0.04	0.70
NESDA_EUR	2,332	0.04	0.13
PSYCOLAUS_EUR	1,631	0.03	0.51
YFS_EUR	1,551	0.03	0.92
SOL_HISP	7,958	0.08	6.7e-03
**Stage 2 CPMA**	**16,111**	**0.03**	**0.02**
**Stage 1 + 2 CPMA**	**42,162**	**0.03**	**3.1e-08**

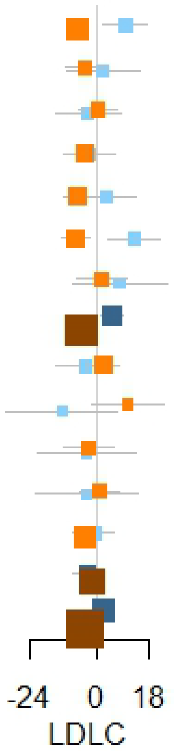

D.

Interaction of rs11949029 and SOCS (2df) on LDLC

Study	N	EAF	P-value
JHS_AFR	1,554	0.0404	8.0e-04
WHI_AFR	7,415	0.0344	3.0e-03
**Stage 1 CPMA**	**8,969**	**0.0354**	**2.1e-05**
SOL_HISP	7,958	0.0059	1.6e-04
**Stage 2 CPMA**	**7,958**	**0.0059**	**1.6e-04**
**Stage 1 + 2 CPMA**	**16,927**	**0.0215**	**2.3e-08**

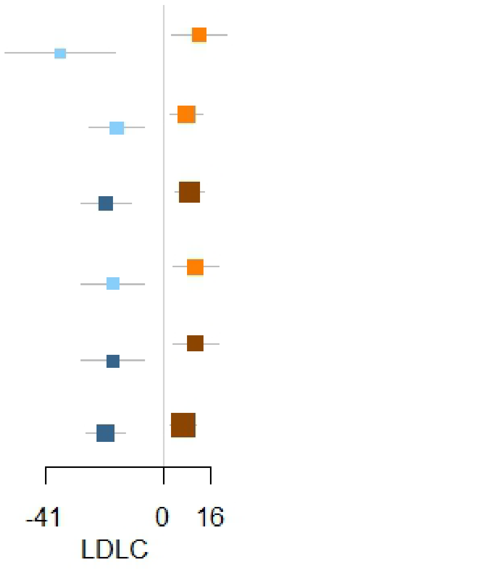

E.

Interaction of rs4562311 and ANXT (2df) on TG

Study	N	EAF	P-value
BAEPENDI_BRZ	750	0.06	1.8e-08
CARDIA_AFR	807	0.31	5.6e-03
MESA_AFR	1,607	0.32	0.21
**Stage 1 CPMA**	**3,164**	**0.25**	**5.5e-11**
GENESTAR_AFR	1,069	0.29	0.67
KORA_F4_EUR	2,434	0.01	0.18
**Stage 2 CPMA**	**3,503**	**0.1**	**0.75**
**Stage 1 + 2 CPMA**	**6,667**	**0.17**	**8.5e-09**

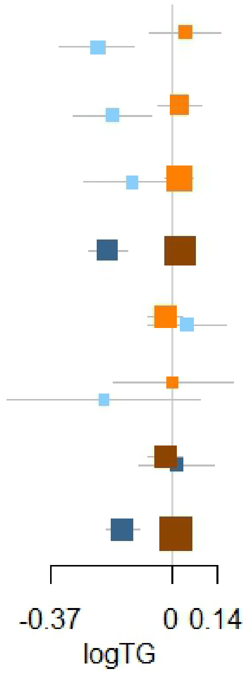

F.

Interaction of rs59808825 and ANXT (2df) on LDLC

Study	N	EAF	P-value
BAEPENDI_BRZ	743	0.04	2.1e-07
PELOTAS_BRZ	2,693	0.04	0.01
**Stage 1 CPMA**	**3,436**	**0.04**	**1.9e-07**
NESDA_EUR	2,537	0.02	3.8e-03
**Stage 2 CPMA**	**2,537**	**0.02**	**3.8e-03**
**Stage 1 + 2 CPMA**	**5,973**	**0.03**	**8.8e-09**

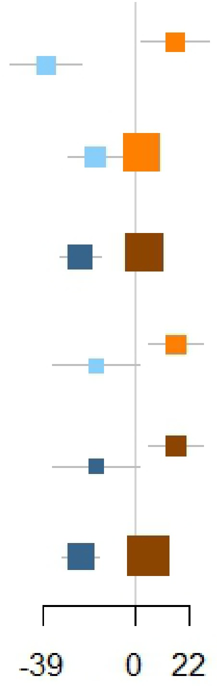

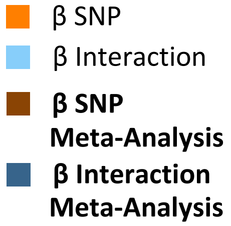

G.

Interaction of rs73597733 and DEPR (2df) on HDLC

Study	N	EAF	P-value
HANDLS_AFR	928	0.04	2.6e-06
JHS_AFR	1,245	0.04	0.04
WHI_AFR	7,415	0.03	2.2e-04
**Stage 1 AFR**	**9,588**	**0.04**	**9.6e-10**
HRS_AFR	1,646	0.03	0.35
**Stage 2 AFR**	**1,646**	**0.03**	**0.35**
**Stage 1 + 2 AFR**	**11,234**	**0.04**	**1.0e-08**
SOL_HISP	11,524	0.01	0.71
**Stage 1 + 2 CPMA**	**22,758**	**0.02**	**2.7e-05**

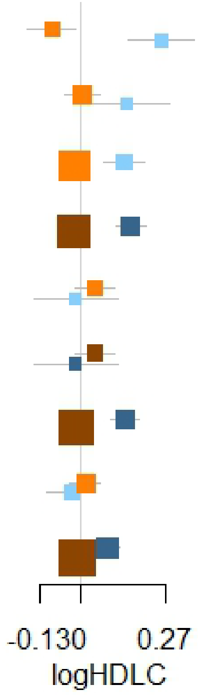

H. **Interaction of rs11702544 and DEPR (2df) on HDLC**

Study	N	EAF	P-value
Stage 1 AFR	14,499	0.45	0.16
Stage 1 ASN	748	0.21	0.64
Stage 1 BRZ	3,464	0.41	0.61
Stage 1 EUR	34,079	0.42	3.3e-07
Stage 1 HISP	4,453	0.29	0.33
**Stage 1 CPMA**	**57,243**	**0.41**	**1.1e-07**
Stage 2 AFR	1,077	0.47	0.77
Stage 2 ASN	982	0.23	0.65
Stage 2 EUR	30,338	0.4	0.10
Stage 2 HISP	11,524	0.3	0.42
**Stage 2 CPMA**	**42,939**	**0.38**	**0.08**
**Stage 1 + 2 CPMA**	**100,182**	**0.4**	**2.3e-08**

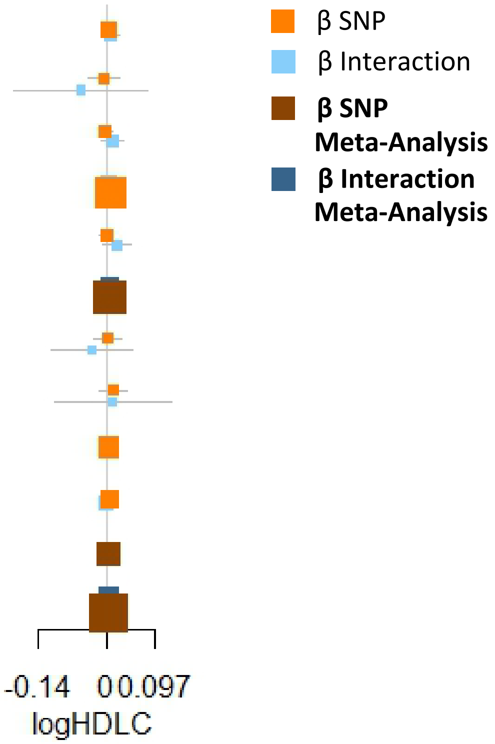

Study Descriptions

AGES (Age Gene/Environment Susceptibility Reykjavik Study): The AGES Reykjavik study originally comprised a random sample of 30,795 men and women born in 1907-1935 and living in Reykjavik in 1967. A total of 19,381 people attended, resulting in a 71% recruitment rate. The study sample was divided into six groups by birth year and birth date within month. One group was designated for longitudinal follow up and was examined in all stages; another was designated as a control group and was not included in examinations until 1991. Other groups were invited to participate in specific stages of the study. Between 2002 and 2006, the AGESReykjavik study re-examined 5,764 survivors of the original cohort who had participated before in the Reykjavik Study.

ARIC (Atherosclerosis Risk in Communities): The ARIC study is a population-based prospective cohort study of cardiovascular disease sponsored by the National Heart, Lung, and Blood Institute (NHLBI). ARIC included 15,792 individuals, predominantly European American and African American, aged 45-64 years at baseline (1987-89), chosen by probability sampling from four US communities. Cohort members completed three additional triennial follow-up examinations, a fifth exam in 2011-2013, a sixth exam in 2016-2017, a seventh exam in 2018-2019, an eighth exam in 2020, and a ninth exam in 2022. The ARIC study has been described in detail previously:

1. Wright JD, Folsom AR, Coresh J, et al. The ARIC (Atherosclerosis Risk In Communities) Study: JACC Focus Seminar 3/8. J Am Coll Cardiol. 2021 Jun 15;77(23):2939-2959

Baependi Heart Study (Brazil): The Baependi Heart Study, is an ongoing family-based cohort conducted in a rural town of the state of Minas Gerais. The study has enrolled approximate 2,200 individuals (over 10% of the town's adult population) and 10-year follow up period of longitudinal data. Briefly, probands were selected at random across 11 out of the 12 census districts in Baependi. After enrolment, the proband's first-degree (parents, siblings, and offspring), second-degree (half-siblings, grandparents/grandchildren, uncles/aunts, nephews/nieces, and double cousins), and third-degree (first cousins, great uncles/aunts, and great nephews/nieces) relatives, and his/her respective spouse's relatives resident both within Baependi (municipal and rural area) and surrounding towns were invited to participate. Only individuals age 18 and older were eligible to participate in the study. The study is conducted from a clinic/office in an easily accessible sector of the town, where the questionnaires were completed. A broad range of phenotypes ranging from cardiovascular, neurocognitive, psychiatric, imaging, physiologic and several layers of endophenotypes like metabolomics and lipidomics have been collected throughout the years Details about follow-up visits and available data can be found in the cohort profile paper (PMID: 18430212). DNA samples were genotyped using the Affymetrix 6.0 genechip. After quality control, the data were prephased using SHAPEIT and imputed using IMPUTE2 based on 1000 Genomes haplotypes.

BES (Beijing Eye Study): The Beijing Eye Study is a population-based study that assesses the associated and risk factors of ocular and general diseases in a Chinese population. The study was initialized in 2001 and collected data from 4439 subjects aged ≥ 40 years and living in seven communities in the Beijing area. Three of these communities were located in a rural district and four were located in an urban district. The BES was followed-up in 2006, with 3251 of the original subjects participating, and in 2011, with 2695 subjects returning for the follow-up examination.

At the examinations in 2006 and 2011, trained research staffs asked the subjects questions from a standard questionnaire providing information on the family status, level of education, income, quality of life, psychic depression, physical activity, and known major systemic diseases. Fasting blood samples were taken for measurement of concentrations of substances such as blood lipids, glucose, and glycosylated hemoglobin. Individuals were classified as self-reported non-smokers or self-reported current smokers. Alcohol consumption habits based on number of drinks per day were collected. Physical activity was assessed in questions on the number of hours per day and number of days per week spent on intensively or moderately performed sport activities, spent on walking, on riding a bicycle, and spent on sitting. All variables used in analyses were taken from examinations in 2006 or in 2011. The BES subjects were genotyped on two arrays, Illumina Human610-Quad (N = 832) and Illumina OmniExpress (N = 814).

CARDIA (Coronary Artery Risk Development in Young Adults): CARDIA is a longitudinal multicenter study with 5,115 Black and White participants 18-30 years of age at baseline, recruited from four centers in 1985-1986. The recruitment was done from the total community in Birmingham, AL; from selected census tracts in Chicago, IL and Minneapolis, MN; and from the Kaiser Permanente health plan membership in Oakland, CA. The details of the study design for the CARDIA study have been previously published.[1] Ten examinations have been completed since initiation of the study, respectively in the years 0, 2, 5, 7, 10, 15, 20, 25, 30, and 35. Written informed consent was obtained from participants at each examination, and all study protocols were approved by the institutional review boards of the participating institutions.

Age and race were self-reported at baseline using standardized questionnaires, as were use of cholesterol-lowering medication, and smoking status (current, former, or never). All participants were asked to fast for 12 hours before each clinic visit. Lipid measures were performed on plasma blood samples drawn from the antecubital vein and stored at −70° C until analyzed. Plasma total cholesterol, HDL-cholesterol, and triglyceride concentrations were measured using enzymatic methods;[2] HDL-cholesterol concentrations were measured after dextran-sulfate-magnesium precipitation of other lipoproteins.[3] LDL-cholesterol concentrations were estimated with the Friedewald equation for individuals with fasting triglyceride values less than 400 mg/dL.[4] The test-retest correlations for total cholesterol, HDL-cholesterol, LDL-cholesterol, and triglycerides were 0.98 to 0.99.[5]

1. Friedman GD, Cutter GR, Donahue RP, Hughes GH, Hully SB, Jacobs DR Jr., Liu K, Savage PJ. CARDIA: study design, recruitment, and some characteristics of the examined subjects. J Clin Epidemiol. 1988;41:1105–1116.

2. Warnick GR. Enzymatic methods for quantification of lipoprotein lipids. Methods Enzymol. 1986;129:101–123.

3. Warnick GR, Benderson J, Albers JJ. Dextran sulfate-Mg2+ precipitation procedure for quantitation of high-density-lipoprotein cholesterol. Clin Chem. 1982;28:1379–1388.

4. Friedewald WT, Levy RI, Fredrickson DS. Estimation of the concentration of low-density lipoprotein cholesterol in plasma, without use of the preparative ultracentrifuge. Clin Chem. 1972;18:499–502.

5. Gross M, Steffes M, Jacobs DR Jr., Yu X, Lewis L, Lewis CE, Loria CM. Plasma F2-isoprostanes and coronary artery calcification: the CARDIA Study. Clin Chem. 2005;51:125–131.

CHS (Cardiovascular Health Study): CHS is a population-based cohort study of risk factors for cardiovascular disease in adults 65 years of age or older conducted across four field centers (1). The original predominantly European ancestry cohort of 5,201 persons was recruited in 1989-1990 from random samples of the Medicare eligibility lists and an additional predominately African-American cohort of 687 persons was enrolled in 1992-93 for a total sample of 5,888. Blood samples were drawn from all participants at their baseline examination and DNA was subsequently extracted from available samples. European ancestry participants were excluded from the GWAS study sample due to prevalent coronary heart disease, congestive heart failure, peripheral vascular disease, valvular heart disease, stroke, or transient ischemic attack at baseline. After QC, genotyping was successful for 3271 European ancestry and 823 African-American participants. CHS was approved by institutional review committees at each site and individuals in the present analysis gave informed consent including consent to use of genetic information for the study of cardiovascular disease.

1. Fried LP, Borhani NO, Enright P, Furberg CD, Gardin JM, Kronmal RA, et al. The Cardiovascular Health Study: design and rationale. Ann Epidemiol 1991; 1:263-76.

CoLaus|PsyCoLaus: The CoLaus|PsyCoLaus study is a population-based cohort of 6734 participants from Lausanne (Switzerland) aged 35-75 at recruitment (2003-2006). This study collects detailed and standardized phenotypes to investigate biological, genetic, and environmental determinants of cardiovascular disease and their association with mental disorders. The main aims of the study were: 1) to better understand the epidemiology of cardiovascular risk factors and diseases in the Swiss population; 2) to discover new genetic determinants of these conditions; and 3) to determine the nature of the associations between cardiovascular disease and mental disorders.

Since then, three follow-up surveys have been completed (2009-2012, 2014-2016, 2018-2020) and a fourth one is ongoing since 2022. A detailed description of the participants' selection, inclusion criteria and methods used can be found in the following manuscripts:

1. Firmann M, Mayor V, Marques Vidal P, Bochud M, Pécoud A, Hayoz D, Paccaud F, Preisig M, Song KS, Yuan X, et al. The CoLaus study: a population-based study to investigate the genetic determinants of cardiovascular risk factors and metabolic syndrome. BMC Cardiovascular Disorders. 2008; 8:6. PMID: 18366642; PMCID: *PMC2311269*; DOI: 10.1186/1471-2261-8-6.

2. Preisig M, Waeber G, Vollenweider P, Bovet P, Rothen S, Vandeleur C, et al. The PsyCoLaus study: methodology and characteristics of the sample of a population-based survey on psychiatric disorders and their association with genetic and cardiovascular risk factors. BMC psychiatry. 2009;9:9. PMID: 19292899 PMCID: PMC2667506 DOI: 10.1186/1471-244X-9-9.

DR's EXTRA (Dose-Responses to Exercise Training): The Dose-Responses to Exercise Training (DR's EXTRA) Study is a 4-year RCT on the effects of regular physical exercise and

healthy diet on endothelial function, atherosclerosis and cognition in a randomly selected population sample (n=3000) of Eastern Finnish men and women, identified from the national population register, aged 55-74 years. Of the eligible sample, 1410 individuals were randomized into one of the 6 groups: aerobic exercise, resistance exercise, diet, combined aerobic exercise and diet, combined resistance exercise and diet, or reference group following baseline assessments. During the four year intervention the drop-out rate was 15%.

ERF (Erasmus Rucphen Family study): Erasmus Rucphen Family is a family based study that includes inhabitants of a genetically isolated community in the South-West of the Netherlands, studied as part of the Genetic Research in Isolated Population (GRIP) program.[1,2] The goal of the study is to identify the risk factors in the development of complex disorders. Study population includes approximately 3,000 individuals who are living descendants of 22 couples who lived in the isolate between 1850 and 1900 and had at least six children baptized in the community church. All data were collected between 2002 and 2005. All participants gave informed consent, and the Medical Ethics Committee of the Erasmus University Medical Centre approved the study.

1. Pardo, Luba M., et al. "The effect of genetic drift in a young genetically isolated population." Annals of human genetics 69.3 (2005): 288-295.

2. Aulchenko, Yurii S., et al. "Linkage disequilibrium in young genetically isolated Dutch population." European Journal of Human Genetics 12.7 (2004): 527.

FHS (Framingham Heart Study): FHS began in 1948 with the recruitment of an original cohort of 5,209 men and women (mean age 44 years; 55 percent women). In 1971 a second generation of study participants was enrolled; this cohort (mean age 37 years; 52% women) consisted of 5,124 children and spouses of children of the original cohort. A third generation cohort of 4,095 children of offspring cohort participants (mean age 40 years; 53 percent women) was enrolled in 2002-2005 and are seen every 4 to 8 years. Details of study designs for the three cohorts are summarized elsewhere. At each clinic visit, a medical history was obtained with a focus on cardiovascular content, and participants underwent a physical examination including measurement of height and weight from which BMI was calculated. For this study, lipid measurements were used from the first exam of the 2nd generation (1971-1975) and the 3rd generation (2002-2005) cohorts. Fasting levels of total cholesterol, high-density lipoprotein cholesterol, and triglycerides were measured using standard enzymatic methods in accordance with LRC protocols. LDL cholesterol was calculated using the Friedewald formula.

GeneSTAR (Genetic Studies of Atherosclerosis Risk): GeneSTAR is a family-based prospective study of more than 4000 participants begun in 1983 to determine phenotypic and genetic causes of premature cardiovascular disease. Families were identified from 1983-2006 from probands with a premature coronary disease event prior to 60 years of age who were identified at the time of hospitalization in any of 10 hospitals in the Baltimore, Maryland area. Their apparently healthy 30-59 year old siblings without known coronary disease were recruited and screened between 1983 and 2006. From 2003-2006, adult offspring over 21 years of age of all participating siblings and probands, as well as the coparents of the offspring were recruited and screened. Genotyping was performed in 3,232 participants on the Illumina 1Mv1_c platform. Demographic information including education level was self-reported by participants. Medical history including medication use was assessed by the study nurse or physician.

GENOA (Genetic Epidemiology Network of Arteriopathy): GENOA is one of four networks in the NHLBI Family-Blood Pressure Program (FBPP). [The FBPP Investigators, 2002; Daniels et al., 2004] GENOA's long-term objective is to elucidate the genetics of target organ complications of hypertension, including both atherosclerotic and arteriosclerotic complications involving the heart, brain, kidneys, and peripheral arteries. The longitudinal GENOA Study recruited European-American and African-American sibships with at least 2 individuals with clinically diagnosed essential hypertension before age 60 years. All other members of the sibship were invited to participate regardless of their hypertension status. Participants were diagnosed with hypertension if they had either 1) a previous clinical diagnosis of hypertension by a physician with current anti-hypertensive treatment, or 2) an average systolic blood pressure ≥ 140 mm Hg or diastolic blood pressure ≥ 90 mm Hg based on the second and third readings at the time of their clinic visit. Exclusion criteria were secondary hypertension, alcoholism or drug abuse, pregnancy, insulin-dependent diabetes mellitus, or active malignancy. During the first exam (1995-2000), 1,583 European Americans from Rochester, MN and 1,854 African Americans from Jackson, MS were examined. Between 2000 and 2005, 1,241 of the European Americans and 1,482 of the African Americans returned for a second examination. In a GENOA follow-up study (2009-2011), 752 African American participated in a third examination. Depression was only evaluated in the third examination; thus, the analysis for this project includes African American participants who participated in this examination. Because African-American probands for GENOA were recruited through the Atherosclerosis Risk in Communities (ARIC) Jackson field center participants, we excluded ARIC participants from GENOA analysis.

1. The FBPP Investigators. Multi-center genetic study of hypertension: The Family Blood Pressure Program (FBPP). Hypertension 2002;39:3-9. PubMed PMID: 11799070.

2. Daniels PR, Kardia SL, Hanis CL, Brown CA, Hutchinson R, Boerwinkle E, Turner ST; Genetic Epidemiology Network of Arteriopathy study. Familial aggregation of hypertension treatment and control in the Genetic Epidemiology Network of Arteriopathy (GENOA) study. Am J Med. 2004;116:676-81. PubMed PMID: 15121494.

HANDLS (Healthy Aging in Neighborhoods of Diversity across the Life Span): HANDLS is a community-based, longitudinal epidemiologic study examining the influences of race and socioeconomic status (SES) on the development of age-related health disparities among a sample of socioeconomically diverse African Americans and whites. This unique study will assess over a 20-year period physical parameters and also evaluate genetic, biologic, demographic, and psychosocial, parameters of African American and white participants in higher and lower SES to understand the driving factors behind persistent black-white health disparities in overall longevity, cardiovascular disease, and cognitive decline. The study recruited 3,722 participants from Baltimore, MD with a mean age of 47.7 years, 2,200 African Americans and 1,522 whites, with 41% reporting household incomes below the 125% poverty delimiter.

Genotyping was done on a subset of self-reporting African American participants by the Laboratory of Neurogenetics, National Institute on Aging, National Institutes of Health (NIH). A larger genotyping effort included a small subset of self-reporting European ancestry samples. This research was supported by the Intramural Research Program of the NIH, NIA and the National Center on Minority Health and Health Disparities.

HCHS/SOL (Hispanic Community Health Study/ Study of Latinos): The HCHS/SOL is a community-based cohort study of 16,415 self-identified Hispanic/Latino persons aged 18–74

years and selected from households in predefined census-block groups across four US field centers (in Chicago, Miami, the Bronx, and San Diego). The census-block groups were chosen to provide diversity among cohort participants with regard to socioeconomic status and national origin or background. The HCHS/SOL cohort includes participants who self-identified as having a Hispanic/Latino background; the largest groups are Central American (n = 1,730), Cuban (n = 2,348), Dominican (n = 1,460), Mexican (n = 6,471), Puerto Rican (n = 2,728), and South American (n = 1,068). The HCHS/SOL baseline clinical examination occurred between 2008 and 2011 and included comprehensive biological, behavioral, and sociodemographic assessments. Consenting HCHS/SOL subjects were genotyped at Illumina on the HCHS/SOL custom 15041502 B3 array. The custom array comprised the Illumina Omni 2.5M array (HumanOmni2.5-8v.1-1) ancestry-informative markers, known GWAS hits and drug absorption, distribution, metabolism, and excretion (ADME) markers, and additional custom content including ~150,000 SNPs selected from the CLM (Colombian in Medellin, Colombia), MXL (Mexican Ancestry in Los Angeles, California), and PUR (Puerto Rican in Puerto Rico) samples in the 1000Genomes phase 1 data to capture a greater amount of Amerindian genetic variation. QA/QC procedures yielded a total of 12,803 unique study participants for imputation and downstream association analyses.

Health ABC (Health, Aging, and Body Composition): Cohort description: The Health ABC study is a prospective cohort study investigating the associations between body composition, weight-related health conditions, and incident functional limitation in older adults. Health ABC enrolled well-functioning, community-dwelling black (n=1281) and white (n=1794) men and women aged 70-79 years between April 1997 and June 1998. Participants were recruited from a random sample of white and all black Medicare eligible residents in the Pittsburgh, PA, and Memphis, TN, metropolitan areas. Participants have undergone annual exams and semi-annual phone interviews. The current study sample consists of 1559 white participants who attended the second exam in 1998-1999 with available genotyping data.

Genotyping: Genotyping was performed by the Center for Inherited Disease Research (CIDR) using the Illumina Human1M-Duo BeadChip system. Samples were excluded from the dataset for the reasons of sample failure, genotypic sex mismatch, and first-degree relative of an included individual based on genotype data. Genotyping was successful in 1663 Caucasians. Analysis was restricted to SNPs with minor allele frequency ≥ 1%, call rate ≥97% and HWE p≥10-6. Genotypes were available on 914,263 high quality SNPs for imputation based on the HapMap CEU (release 22, build 36) using the MACH software (version 1.0.16). A total of 2,543,888 imputed SNPs were analyzed for association with vitamin D levels.

Association analysis: Linear regression models were used to generate cohort-specific residuals of naturally log transformed vitamin D levels adjusted for age, sex, BMI and season defined as summer (June-August), fall (September-November), winter (December to February) and spring (March to May) standardized to have mean 0 and variance of 1. Association between the additively coded SNP genotypes and the vitamin D residuals standardized was assessed using linear regression models. For imputed SNPs, expected number of minor alleles (i.e. dosage) was used in assessing association with the vitamin D residuals.

HRS (Health & Retirement Study): The Health and Retirement Study (HRS) is a longitudinal survey of a representative sample of Americans over the age of 50. The current sample is over 26,000 persons in 17,000 households. Respondents are interviewed every two years about

income and wealth, health and use of health services, work and retirement, and family connections. DNA was extracted from saliva collected during a face-to-face interview in the respondents' homes. These data represent respondents who provided DNA samples and signed consent forms in 2006, 2008, and 2010. Respondents were removed if they had missing genotype or phenotype data.

1. Juster, F. T., Suzman, R. (1995). An Overview of the Health and Retirement Study, Journal of Human Resources 30:Suppl: S7-S56.

2. Sonnega A, Faul JD, Ofstedal MB, Langa KM, Phillips JWR, Weir DR. Cohort Profile: the Health and Retirement Study (HRS). Int. J. Epidemiol. 2014; 43 (2): 576-585. PMID: 24671021.

3. Crimmins, E.M., Guyer H., Langa K.M., Ofstedal M.B., Wallace R.B., and Weir D.R. (2008). Documentation of Physical Measures, Anthropometrics and Blood Pressure in the Health and Retirement Study. HRS Documentation Report DR-011. http://hrsonline.isr.umich.edu/sitedocs/userg/dr-011.pdf

JHS (Jackson Heart Study): The Jackson Heart Study is a longitudinal, community-based observational cohort study investigating the role of environmental and genetic factors in the development of cardiovascular disease in African Americans. Between 2000 and 2004, a total of 5,306 participants were recruited from a tri-county area (Hinds, Madison, and Rankin Counties) that encompasses Jackson, MS. Details of the design and recruitment for the Jackson Heart Study cohort has been previously published.1-3 Briefly, approximately 30% of participants were former members of the Atherosclerosis Risk in Communities (ARIC) study. The remainder were recruited by either 1) random selection from the Accudata list, 2) commercial listing, 3) a constrained volunteer sample, in which recruitment was distributed among defined demographic cells in proportions designed to mirror those in the overall population, or through the Jackson Heart Study Family Study.

1. Wyatt SB, Diekelmann N, Henderson F, Andrew ME, Billingsley G, Felder SH et al. A community-driven model of research participation: the Jackson Heart Study Participant Recruitment and Retention Study. Ethn Dis 2003; 13(4):438-455.

2. Taylor HA, Jr., Wilson JG, Jones DW, et al. Toward resolution of cardiovascular health disparities in African Americans: design and methods of the Jackson Heart Study. Ethn Dis 2005; 15:S6-17.

3. Fuqua SR, Wyatt SB, Andrew ME, et al. Recruiting African-American research participation in the Jackson Heart Study: methods, response rates, and sample description. Ethn Dis 2005; 15:S6-29.

KORA (Cooperative Health Research in the Augsburg Region): The KORA study is a series of independent population-based epidemiological surveys of participants living in the region of Augsburg, Southern Germany. All survey participants are residents of German nationality identified through the registration office and were examined in 1994/95 (KORA S3) and 1999/2001 (KORA S4). In the KORA S3 and S4 studies 4,856 and 4,261 subjects have been examined implying response rates of 75% and 67%, respectively. 3,006 subjects participated in a 10-year follow-up examination of S3 in 2004/05 (KORA F3), and 3080 of S4 in 2006/2008

(KORA F4). The age range of the participants was 25 to 74 years at recruitment. Informed consent has been given by all participants. The study has been approved by the local ethics committee. Individuals for genotyping in KORA F3 and KORA F4 were randomly selected and these genotypes are taken for the analysis of the phenotypes in KORA S3 and KORA S4.

LBC1936 (Lothian Birth Cohort 1936): LBC1936 consists of 1091 (548 male) relatively healthy individuals who underwent cognitive and medical testing at a mean age of 69.6 years (SD = 0.8). They were born in 1936, most took part in the Scottish Mental Survey of 1947, and almost all lived independently in the Lothian region of Scotland.[1,2]

1. Deary IJ, Gow AJ, Pattie A, Starr JM. Cohort profile: the Lothian Birth Cohorts of 1921 and 1936. Int J Epidemiol 2012;41:1576-1584.

2. Taylor AM, Pattie A, Deary IJ. Cohort Profile Update: The Lothian Birth Cohorts of 1921 and 1936. Int J Epidemiol 2018;47:1042-1042r

Lifelines (Netherlands Biobank): Lifelines (https://lifelines.nl/) is a multi-disciplinary prospective population-based cohort study using a unique three-generation design to examine the health and health-related behaviors of 167,729 persons living in the North East region of The Netherlands. It employs a broad range of investigative procedures in assessing the biomedical, socio-demographic, behavioral, physical and psychological factors which contribute to the health and disease of the general population, with a special focus on multimorbidity and complex genetics. In addition, the Lifelines project comprises a number of cross-sectional sub-studies, which investigate specific age-related conditions. These include investigations into metabolic and hormonal diseases, including obesity, cardiovascular and renal diseases, pulmonary diseases and allergy, cognitive function and depression, and musculoskeletal conditions. Recruitment has been going on since the end of 2006, and over 130,000 participants had been included by April 2013. At the baseline examination, the participants in the study were asked to fill in a questionnaire (on paper or online) before the first visit. During the first and second visit, the first or second part of the questionnaire, respectively, are checked for completeness, a number of investigations are conducted, and blood and urine samples are taken. Lifelines is a facility that is open for all researchers. Information on application and data access procedure is summarized on www.lifelines-biobank.com. (Scholtens S, Smidt N, Swertz MA, Bakker SJ, Dotinga A, Vonk JM, et al. Cohort Profile: LifeLines, a three-generation cohort study and biobank. Int J Epidemiol. 2014 Dec 14.)

Lifelines group authors (genetics): Behrooz Z Alizadeh,[1] H Marike Boezen,[1] Lude Franke,[2] Pim van der Harst,[3] Gerjan Navis,[4] Marianne Rots,[5] Harold Snieder,[1] Morris Swertz,[2] Bruce HR Wolffenbuttel,[6] Cisca Wijmenga.[2]

[1] Department of Epidemiology, University of Groningen, University Medical Center Groningen, The Netherlands; [2] Department of Genetics, University of Groningen, University Medical Center Groningen, The Netherlands; [3] Department of Cardiology, University of Groningen, University Medical Center Groningen, The Netherlands; [4] Department of Internal Medicine, Division of Nephrology, University of Groningen, University Medical Center Groningen, The Netherlands; [5] Department of Pathology and Medical Biology, University of Groningen, University Medical Center Groningen, The Netherlands; [6] Department of Endocrinology, University of Groningen, University Medical Center Groningen, The Netherlands

MESA (Multi-Ethnic Study of Atherosclerosis): The Multi-Ethnic Study of Atherosclerosis (MESA) is a study of the characteristics of subclinical cardiovascular disease and the risk factors that predict progression to clinically overt cardiovascular disease or progression of the subclinical disease. MESA consisted of a diverse, population-based sample of an initial 6,814 asymptomatic men and women aged 45-84. 38 percent of the recruited participants were white, 28 percent African American, 22 percent Hispanic, and 12 percent Asian, predominantly of Chinese descent. Participants were recruited from six field centers across the United States: Wake Forest University, Columbia University, Johns Hopkins University, University of Minnesota, Northwestern University and University of California - Los Angeles. Participants are being followed for identification and characterization of cardiovascular disease events, including acute myocardial infarction and other forms of coronary heart disease (CHD), stroke, and congestive heart failure; for cardiovascular disease interventions; and for mortality. The first examination took place over two years, from July 2000 - July 2002. It was followed by four examination periods that were 17-20 months in length. Participants have been contacted every 9 to 12 months throughout the study to assess clinical morbidity and mortality.

1. Bild DE, Bluemke DA, Burke GL, Detrano R, Diez Roux AV, Folsom AR, Greenland P, Jacob DR Jr, Kronmal R, Liu K, Nelson JC, O'Leary D, Saad MF, Shea S, Szklo M, Tracy RP. Multi-ethnic study of atherosclerosis: objectives and design. Am J Epidemiol. 2002 Nov 1;156(9):871-81. PubMed PMID: 12397006.

NEO (The Netherlands Epidemiology of Obesity study): The NEO was designed for extensive phenotyping to investigate pathways that lead to obesity-related diseases. The NEO study is a population-based, prospective cohort study that includes 6,671 individuals aged 45–65 years, with an oversampling of individuals with overweight or obesity. At baseline, information on demography, lifestyle, and medical history have been collected by questionnaires. In addition, samples of 24-h urine, fasting and postprandial blood plasma and serum, and DNA were collected. Genotyping was performed using the Illumina HumanCoreExome chip, which was subsequently imputed to the 1000 genome reference panel. Participants underwent an extensive physical examination, including anthropometry, electrocardiography, spirometry, and measurement of the carotid artery intima-media thickness by ultrasonography. In random subsamples of participants, magnetic resonance imaging of abdominal fat, pulse wave velocity of the aorta, heart, and brain, magnetic resonance spectroscopy of the liver, indirect calorimetry, dual energy X-ray absorptiometry, or accelerometry measurements were performed. The collection of data started in September 2008 and completed at the end of September 2012. Participants are currently being followed for the incidence of obesity-related diseases and mortality.

NESDA (Netherlands Study of Depression and Anxiety): NESDA is a multi-center study designed to examine the long-term course and consequences of depressive and anxiety disorders (http://www.nesda.nl)[1]. NESDA included both individuals with depressive and/or anxiety disorders and controls without psychiatric conditions. Inclusion criteria were age 18-65 years and self-reported western European ancestry while exclusion criteria were not being fluent in Dutch and having a primary diagnosis of another psychiatric condition (psychotic disorder, obsessive compulsive disorder, bipolar disorder, or severe substance use disorder).

1. Penninx, B.W. et al. Cohort profile of the longitudinal Netherlands Study of Depression and Anxiety (NESDA) on etiology, course and consequences of depressive and anxiety disorders. J Affect Disord. 2021 May 15;287:69-77.

Pelotas Birth Cohort Study (The 1982 Pelotas Birth Cohort Study, Brazil): The maternity hospitals in Pelotas, a southern Brazilian city (current population ~330,000), were visited daily in the year of 1982. The 5,914 liveborns whose families lived in the urban area were examined and their mothers interviewed. Information was obtained for more than 99% of the livebirths. These subjects have been followed-up at the following mean ages: 11.3 months (all children born from January to Abril 1982; n=1457), 19.4 months (entire cohort; n=4934), 43.1 months (entire cohort; n=4742), 13.1 years (random subsample; n=715), 14.7 years (systematic subsample; n=1076); 18.2 (male cohorts attending to compulsory Army recruitment examination; n=2250), 18.9 (systematic subsample; n=1031), 22.8 years (entire cohort; n=4297) and 30.2 years (entire cohort; n=3701). Details about follow-up visits and available data can be found in the two Cohort Profile papers (PMID: 16373375 and 25733577). DNA samples (collected at the mean age of 22.8 years) were genotyped for ~2.5 million of SNPs using the Illumina HumanOmni2.5-8v1 array (which includes autosomal, X and Y chromosomes, and mitochondrial variants). After quality control, the data were prephased using SHAPEIT and imputed using IMPUTE2 based on 1000 Genomes haplotypes.

RS (Rotterdam Study): The Rotterdam Study is a prospective, population-based cohort study among individuals living in the well-defined Ommoord district in the city of Rotterdam in The Netherlands.[1] The aim of the study is to determine the occurrence of cardiovascular, neurological, ophthalmic, endocrine, hepatic, respiratory, and psychiatric diseases in elderly people. The cohort was initially defined in 1990 among 7,983 persons, aged 55 years and older, who underwent a home interview and extensive physical examination at the baseline and during follow-up rounds every 3-4 years (RS-I). Cohort was extended in 2000/2001 (RS-II, 3,011 individuals aged 55 years and older) and 2006/2008 (RS-III, 3,932 subjects, aged 45 and older; since 2016, the cohort was expanded to include persons aged 40 years and older).[1] The Rotterdam Study has been approved by the Medical Ethics Committee of the Erasmus MC (registration number MEC 02.1015) and by the Dutch Ministry of Health, Welfare and Sport (Population Screening Act WBO, license number 1071272-159521-PG). The Rotterdam Study Personal Registration Data collection is filed with the Erasmus MC Data Protection Officer under registration number EMC1712001. The Rotterdam Study has been entered into the Netherlands National Trial Register (NTR; www.trialregister.nl) and into the WHO International Clinical Trials Registry Platform (ICTRP; www.who.int/ictrp/network/primary/en/) under shared catalogue number NTR6831. All participants provided written informed consent to participate in the study and to have their information obtained from treating physicians.

1. Ikram, M.A., Kieboom, B.C., Brouwer, W.P. et al. The Rotterdam Study. Design update and major findings between 2020 and 2024. Eur J Epidemiol (2024). https://doi.org/10.1007/s10654-023-01094

SP2-1M / SP2-610 (Singapore Prospective Study Program): The SP2 (Singapore Prospective Study Program) is a population-based study of diabetes and cardiovascular disease in Singapore. It first surveyed subjects (Chinese, Malay and Indian) from four cross-sectional studies that were conducted in Singapore between 1982 and 1998. Subjects were between the ages of 24-95 years and represented a random sample of the Singapore population. Subjects

were re-visited between 2003 and 2007. Among the 10,747 individuals who were eligible, 5,157 subjects completed a questionnaire and the subsequent clinical examinations. Of the 5,517 subjects, 2,434 Chinese were genotyped on a combination of Illumina 610, 1M and 550 arrays. Fasting HDL-C, TC and TG were measured by an automated analyzer autoanalyzer (ADVIA 2400, Bayer Diagnostics). LDL-C was calculated from Friedewald formula. Data from this revisit were utilized for this study.

1. Nang EE, Khoo CM, Tai ES, Lim SC, Tavintharan S, Wong TY, Heng D, Lee J. Is there a clear threshold for fasting plasma glucose that differentiates between those with and without neuropathy and chronic kidney disease?: the Singapore Prospective Study Program. Am J Epidemiol. 2009 Jun 15;169(12):1454-62. doi: 10.1093/aje/kwp076. Epub 2009 Apr 30. PMID: 19406920.

2. Tan KHX, Tan LWL, Sim X, Tai ES, Lee JJ, Chia KS, van Dam RM. Cohort Profile: The Singapore Multi-Ethnic Cohort (MEC) study. Int J Epidemiol. 2018 Jun 1;47(3):699-699j. doi: 10.1093/ije/dyy014. PMID: 29452397.

SHIP (Study of Health in Pomerania): The Study of Health In Pomerania (SHIP) is a prospective longitudinal population-based cohort study in Mecklenburg-West Pomerania assessing the prevalence and incidence of common diseases and their risk factors (PMID: 20167617). SHIP encompasses the two independent cohorts SHIP-START and SHIP-TREND. Participants aged 20 to 79 with German citizenship and principal residency in the study area were recruited from a random sample of residents living in the three local cities, 12 towns as well as 17 randomly selected smaller towns. Individuals were randomly selected stratified by age and sex in proportion to population size of the city, town or small towns, respectively. A total of 4,308 participants were recruited between 1997 and 2001 in the SHIP-START cohort. Between 2008 and 2012 a total of 4,420 participants were recruited in the SHIP-TREND cohort. Individuals were invited to the SHIP study centre for a computer-assisted personal interviews and extensive physical examinations. The study protocol was approved by the medical ethics committee of the University of Greifswald. Oral and written informed consents was obtained from each of the study participants.

Genome-wide SNP-typing was performed using the Affymetrix Genome-Wide Human SNP Array 6.0 or the Illumina Human Omni 2.5 array (SHIP-TREND samples). Array processing was carried out in accordance with the manufacturer’s standard recommendations. Genotypes were determined using GenomeStudio Genotyping Module v1.0 (GenCall) for SHIP-TREND and the Birdseed2 clustering algorithm for SHIP-START. Imputation of genotypes in SHIP-START and SHIP-TREND was performed with the software IMPUTE v2.2.2 based on 1000 Genomes release March 2012.

SWHS/SMHS (Shanghai Women's Health Study/ Shanghai Men's Health Study): The Shanghai Women's Health Study (SWHS) is an ongoing population-based cohort study of approximately 75,000 women who were aged 40-70 years at study enrollment and resided in in urban Shanghai, China; 56,832 (75.8%) provided a blood samples. Recruitment for the SWHS was initiated in 1997 and completed in 2000. The self-administered questionnaire includes information on demographic characteristics, disease and surgery histories, personal habits (such as cigarette smoking, alcohol consumption, tea drinking, and ginseng use), menstrual history, residential history, occupational history, and family history of cancer.

The Shanghai Men's Health Study (SMHS) is an ongoing population-based cohort study of 61,480 Chinese men who were aged between 40 and 74 years, were free of cancer at enrollment, and lived in urban Shanghai, China; 45,766 (74.4%) provided a blood samples. Recruitment for the SMHS was initiated in 2002 and completed in 2006. The self-administered questionnaire includes information on demographic characteristics, disease and surgery histories, personal habits (such as cigarette smoking, alcohol consumption, tea drinking, and ginseng use), residential history, occupational history, and family history of cancer.

Genotyping and imputation: Genomic DNA was extracted from buffy coats by using a Qiagen DNA purification kit (Valencia, CA) or Puregene DNA purification kit (Minneapolis, MN) according to the manufacturers' instructions and then used for genotyping assays. The GWAS genotyping was performed using the Affymetrix Genome-Wide Human SNP Array 6.0 (Affy6.0) platform or Illumina 660, following manufacturers' protocols. After sample quality control, we exclude SNPs with 1) MAF <0.01; 2) call rate <95%; 2) bad genotyping cluster; and 3) concordance rate <95% among duplicated QC samples. Genotypes were imputed using the program MACH (http://www.sph.umich.edu/csg/abecasis/MACH/download/), which determines the probable distribution of missing genotypes conditional on a set of known haplotypes, while simultaneously estimating the fine-scale recombination map. Phased autosome SNP data from HapMap Phase II Asians (release 22) were used as the reference. To test for associations between the imputed SNP data with BMI, linear regression (additive model) was used, in which SNPs were represented by the expected allele count, an approach that takes into account the degree of uncertainty of genotype imputation (http://www.sph.umich.edu/csg/abecasis/MACH/download/).

The lipid profiles were measured at Vanderbilt Lipid Laboratory. Total cholesterol, high-density lipoprotein (HDL) cholesterol, and triglycerides (TG) were measured using an ACE Clinical Chemistry System (Alfa Wassermann, Inc, West Caldwell, NJ). Low-density lipoprotein (LDL) cholesterol levels were calculated by using the Friedwald equation. The levels of LDL cholesterol were directly measured using an ACE Clinical Chemistry System for subjects with TG levels ≥ 400 mg/dL. Fasting status was defined as an interval between the last meal and blood draw of 8 hours or longer.

TRAILS (Tracking Adolescents' Individual Lives Survey): TRAILS is a prospective cohort study of Dutch adolescents and young adults, with bi- or triennial measurements from age 11 onwards, which started in 2001. TRAILS consists of a general population and a clinical cohort (https://www.trails.nl/en/home). In the population cohort, six assessment waves have been completed to date, at mean ages 11.1 (SD = 0.6), 13.6 (SD = 0.5), 16.3 (SD = 0.7), 19.1 (SD = 0.6), 22.3 (SD = 0.6), and 25.8 (SD = 0.6). Data for the present study were collected in the population cohort only, during the third and fifth assessment waves. The study was approved by the Dutch Central Committee on Research Involving Human Subjects.

WHI (Women's Health Initiative): WHI is a long-term national health study that focuses on strategies for preventing common diseases such as heart disease, cancer and fracture in postmenopausal women. A total of 161,838 women aged 50–79 years old were recruited from 40 clinical centers in the US between 1993 and 1998. WHI consists of an observational study, two clinical trials of postmenopausal hormone therapy (HT, estrogen alone or estrogen plus progestin), a calcium and vitamin D supplement trial, and a dietary modification trial. Study recruitment and exclusion criteria have been described previously. Recruitment was done through mass mailing to age-eligible women obtained from voter registration, driver's license

and Health Care Financing Administration or other insurance lists, with emphasis on recruitment of minorities and older women. Exclusions included participation in other randomized trials, predicted survival < 3 years, alcoholism, drug dependency, mental illness and dementia. For the CT, women were ineligible if they had a systolic BP > 200 mm Hg or diastolic BP > 105 mm Hg, a history of hypertriglyceridemia or breast cancer. Study protocols and consent forms were approved by the IRB at all participating institutions. Socio-demographic characteristics, lifestyle, medical history and self-reported medications were collected using standardized questionnaires at the screening visit.

The genome wide association study (GWAS) non-overlapping samples are composed of a case-control study (WHI Genomics and Randomized Trials Network – GARNET, which included all coronary heart disease, stroke, venous thromboembolic events and selected diabetes cases that happened during the active intervention phase in the WHI HT clinical trials and aged matched controls), women selected to be "representative" of the HT trial (mostly younger white HT subjects that were also enrolled in the WHI memory study - WHIMS) and the WHI SNP Health Association Resource (WHI SHARe), a randomly selected sample of 8,515 African American and 3,642 Hispanic women from WHI. GWAS was performed using Affymetrix 6.0 (WHI-SHARe), HumanOmniExpressExome-8v1_B (WHIMS), Illumina HumanOmni1-Quad v1-0 B (GARNET). Extensive quality control (QC) of the GWAS data included alignment ("flipping") to the same reference panel, imputation to the 1000G data (using the recent reference panel - v3.20101123), identification of genetically related individuals, and computations of principal components (PCs) using methods developed by Price et al. (using EIGENSOFT software 53), and finally the comparison with self-reported ethnicity. After QC and exclusions from analysis protocol, the number of women included in analysis is 4,423 whites for GARNET, 5,202 white for WHIMS, 7,919 for SHARe African American and 3,377 for SHARe Hispanics.

YFS (The Cardiovascular Risk in Young Finns Study): The YFS is a population-based follow up-study started in 1980. The main aim of the YFS is to determine the contribution made by childhood lifestyle, biological and psychological measures to the risk of cardiovascular diseases in adulthood. In 1980, over 3,500 children and adolescents all around Finland participated in the baseline study. The follow-up studies have been conducted mainly with 3-year intervals. The latest 30-year follow-up study was conducted in 2010-11 (ages 33-49 years) with 2,063 participants. The study was approved by the local ethics committees (University Hospitals of Helsinki, Turku, Tampere, Kuopio and Oulu) and was conducted following the guidelines of the Declaration of Helsinki. All participants gave their written informed consent.

Study Acknowledgments

Infrastructure for the CHARGE Consortium is supported in part by the National Heart, Lung, and Blood Institute grant R01HL105756. Infrastructure for the Gene-Lifestyle Working Group in Phase I was supported by the National Heart, Lung, and Blood Institute grant R01HL118305.

AGES (Age Gene/Environment Susceptibility Reykjavik Study): This study has been funded by NIH contract N01-AG012100, HSSN271201200022C, the NIA Intramural Research Program, an Intramural Research Program Award (ZIAEY000401) from the National Eye Institute, an award from the National Institute on Deafness and Other Communication Disorders (NIDCD) Division of Scientific Programs (IAA Y2-DC_1004-02), Hjartavernd (the Icelandic Heart Association), and the Althingi (the Icelandic Parliament). The study is approved by the Icelandic National Bioethics Committee, VSN: 00-063. The researchers are indebted to the participants for their willingness to participate in the study.

ARIC (Atherosclerosis Risk in Communities) Study: The ARIC study has been funded in whole or in part with Federal funds from the National Heart, Lung, and Blood Institute, National Institutes of Health, Department of Health and Human Services, under Contract nos. (75N92022D00001, 75N92022D00002, 75N92022D00003, 75N92022D00004, 75N92022D00005). The authors thank the staff and participants of the ARIC study for their important contributions. Funding was also supported by R01HL087641 and R01HL086694; National Human Genome Research Institute contract U01HG004402; and National Institutes of Health contract HHSN268200625226C. Infrastructure was partly supported by Grant Number UL1RR025005, a component of the National Institutes of Health and NIH Roadmap for Medical Research.

Baependi Heart Study (Brazil): The Baependi Heart Study was supported by Fundação de Amparo a Pesquisa do Estado de São Paulo (FAPESP) (Grant 2013/17368-0), Coordenação de Aperfeiçoamento de Pessoal de Nível Superior (CAPES) and Hospital Samaritano Society (Grant 25000.180.664/2011-35), through Ministry of Health to Support Program Institutional Development of the Unified Health System (SUS-PROADI).

BES (Beijing Eye Study): The Beijing Eye Study has been funded by the National Natural Science Foundation of China (grant 81170890, 81570835) and the Beijing Municipal of Health Reform and Development Project (grant 2019-4).

CARDIA (Coronary Artery Risk Development in Young Adults): The Coronary Artery Risk Development in Young Adults Study (CARDIA) is supported by contracts 75N92023D00002, 75N92023D00003, 75N92023D00004, 75N92023D00005, and 75N92023D00006 from the National Heart, Lung, and Blood Institute (NHLBI). Genotyping was funded as part of the NHLBI Candidate-gene Association Resource (N01-HC-65226) and the NHGRI Gene Environment Association Studies (GENEVA) (U01-HG004729, U01-HG04424, and U01-HG004446). This manuscript has been reviewed by CARDIA for scientific content.

CHS (Cardiovascular Health Study): This Cardiovascular Health Study (CHS) research was supported by NHLBI contracts HHSN268201200036C, HHSN268200800007C, HHSN268200960009C, HHSN268201800001C N01HC55222, N01HC85079, N01HC85080, N01HC85081, N01HC85082, N01HC85083, N01HC85086, 75N92021D00006; and NHLBI grants U01HL080295, U01HL130114, R01HL087652, R01HL105756, R01HL103612, R01HL085251,

and R01HL120393 with additional contribution from the National Institute of Neurological Disorders and Stroke (NINDS). Additional support was provided through R01AG023629 from the National Institute on Aging (NIA). A full list of principal CHS investigators and institutions can be found at CHS-NHLBI.org. The provision of genotyping data was supported in part by the National Center for Advancing Translational Sciences, CTSI grant UL1TR001881, and the National Institute of Diabetes and Digestive and Kidney Disease Diabetes Research Center (DRC) grant DK063491 to the Southern California Diabetes Endocrinology Research Center. The content is solely the responsibility of the authors and does not necessarily represent the official views of the National Institutes of Health.

CoLaus|PsyCoLaus: The CoLaus|PsyCoLaus study was supported by unrestricted research grants from GlaxoSmithKline, the Faculty of Biology and Medicine of Lausanne, the Swiss National Science Foundation (grants 3200B0–105993, 3200B0-118308, 33CSCO-122661, 33CS30-139468, 33CS30-148401, 33CS30_177535, 3247730_204523 and 320030_220190) and the Swiss Personalized Health Network (grant 2018DRI01).

The above first six SNF grants were attributed to Martin Preisig, the seventh to Julien Vaucher and the eight to Carole Clair. One grant from GlaxoSmithKline (no number) was attributed to Martin Preisig for PsyCoLaus, a second grant from GlaxoSmithKline (no number) was attributed to Gérard Waeber and Peter Vollenweider for CoLaus. The grant from the Faculty of Biology and Medicine of Lausanne (no number) was attributed to Peter Vollenweider and the grant from the Swiss Personalized Health Network was attributed to a consortium. The authors would like to express their gratitude to the participants in the Lausanne CoLaus|PsyCoLaus study.

DR's EXTRA (Dose-Responses to Exercise Training): The study was supported by grants from Ministry of Education and Culture of Finland (722 and 627; 2004-2010); Academy of Finland (102318, 104943, 123885, 211119); European Commission FP6 Integrated Project (EXGENESIS), LSHM-CT-2004-005272; City of Kuopio; Juho Vainio Foundation; Finnish Diabetes Association; Finnish Foundation for Cardiovascular Research; Kuopio University Hospital; Päivikki and Sakari Sohlberg Foundation; Social Insurance Institution of Finland 4/26/2010.

ERF (Erasmus Rucphen Family study): The ERF study as a part of EUROSPAN (European Special Populations Research Network) was supported by European Commission FP6 STRP grant number 018947 (LSHG-CT-2006-01947) and also received funding from the European Community's Seventh Framework Programme (FP7/2007-2013)/grant agreement HEALTH-F4-2007-201413 by the European Commission under the programme "Quality of Life and Management of the Living Resources" of 5th Framework Programme (no. QLG2-CT-2002-01254). High-throughput analysis of the ERF data was supported by joint grant from Netherlands Organisation for Scientific Research and the Russian Foundation for Basic Research (NWO-RFBR 047.017.043). We are grateful to all study participants and their relatives, general practitioners and neurologists for their contributions and to P. Veraart for her help in genealogy, J. Vergeer for the supervision of the laboratory work, P. Snijders for his help in data collection and E.M. van Leeuwen for genetic imputation.

FHS (Framingham Heart Study): This research was conducted in part using data and resources from the Framingham Heart Study of the National Heart Lung and Blood Institute of the National Institutes of Health and Boston University School of Medicine. The analyses reflect intellectual

input and resource development from the Framingham Heart Study investigators participating in the SNP Health Association Resource (SHARe) project. This work was partially supported by the National Heart, Lung and Blood Institute's Framingham Heart Study (Contract Nos. N01-HC-25195 and HHSN268201500001I) and its contract with Affymetrix, Inc for genotyping services (Contract No. N02-HL-6-4278). A portion of this research utilized the Linux Cluster for Genetic Analysis (LinGA-II) funded by the Robert Dawson Evans Endowment of the Department of Medicine at Boston University School of Medicine and Boston Medical Center. This research was partially supported by grant R01-DK089256 from the National Institute of Diabetes and Digestive and Kidney Diseases (MPIs: Ingrid B. Borecki, L. Adrienne Cupples, Kari North).

GeneSTAR (Genetic Studies of Atherosclerosis Risk): GeneSTAR was supported by National Institutes of Health grants from the National Heart, Lung, and Blood Institute (HL49762, HL58625, HL071025, U01 HL72518, HL087698, HL092165, HL099747, and K23HL105897), National Institute of Nursing Research (NR0224103), National Institute of Neurological Disorders and Stroke (NS062059), and by grants from the National Center for Research Resources to the Johns Hopkins General Clinical Research Center (M01-RR000052) and the Johns Hopkins Institute for Clinical & Translational Research (UL1 RR 025005).

GENOA (Genetic Epidemiology Network of Arteriopathy): Support for GENOA was provided by the National Heart, Lung and Blood Institute (HL119443, HL118305, HL054464, HL054457, HL054481, HL071917, HL085571, and HL087660) of the National Institutes of Health. Genotyping was performed at the Mayo Clinic (Stephen T. Turner, MD, Mariza de Andrade PhD, Julie Cunningham, PhD). We thank Eric Boerwinkle, PhD and Megan L. Grove from the Human Genetics Center and Institute of Molecular Medicine and Division of Epidemiology, University of Texas Health Science Center, Houston, Texas, USA for their help with genotyping. We would also like to thank the families that participated in the GENOA study.

HANDLS (Healthy Aging in Neighborhoods of Diversity across the Life Span): The Healthy Aging in Neighborhoods of Diversity across the Life Span (HANDLS) study was supported by the Intramural Research Program of the NIH, National Institute on Aging and the National Center on Minority Health and Health Disparities (project # Z01-AG000513 and human subjects protocol number 09-AG-N248). Data analyses for the HANDLS study utilized the high-performance computational resources of the Biowulf Linux cluster at the National Institutes of Health, Bethesda, MD. (http://biowulf.nih.gov; http://hpc.nih.gov)).

HCHS/SOL (Hispanic Community Health Study/ Study of Latinos): The baseline examination of HCHS/SOL was supported by contracts from the National Heart, Lung, and Blood Institute (NHLBI) to the University of North Carolina (N01-HC65233), University of Miami (N01-HC65234), Albert Einstein College of Medicine (N01-HC65235), Northwestern University (N01-HC65236), and San Diego State University (N01-HC65237). The National Institute on Minority Health and Health Disparities, National Institute on Deafness and Other Communication Disorders, National Institute of Dental and Craniofacial Research (NIDCR), National Institute of Diabetes and Digestive and Kidney Diseases (NIDDK), National Institute of Neurological Disorders and Stroke, and NIH Office of Dietary Supplements additionally contributed funding to HCHS/SOL. The Genetic Analysis Center at the University of Washington was supported by NHLBI and NIDCR contracts (HHSN268201300005C AM03 and MOD03). Additional analysis support was provided by 1R01DK101855-01 and 13GRNT16490017. Genotyping was also supported by National Center for Advancing Translational Sciences UL1TR000124 and NIDDK DK063491 to the

Southern California Diabetes Endocrinology Research Center. This research was also supported in part by the Intramural Research Program of the NIDDK, contract no. HHSB268201200054C, and Illumina.

Health ABC (Health, Aging, and Body Composition): Health ABC was funded by the National Institutes of Aging. This research was supported by NIA contracts N01AG62101, N01AG62103, and N01AG62106. The GWAS was funded by NIA grant 1R01AG032098-01A1 to Wake Forest University Health Sciences and genotyping services were provided by the Center for Inherited Disease Research (CIDR). CIDR is fully funded through a federal contract from the National Institutes of Health to The Johns Hopkins University, contract number HHSN268200782096C. This research was supported in part by the Intramural Research Program of the NIH, National Institute on Aging.

HRS (Health & Retirement Study): HRS is supported by the National Institute on Aging (NIA U01AG009740 and R03 AG046389). Genotyping was funded separately by NIA (RC2 AG036495, RC4 AG039029). Our genotyping was conducted by the NIH Center for Inherited Disease Research (CIDR) at Johns Hopkins University. Genotyping quality control and final preparation of the data were performed by the Genetics Coordinating Center at the University of Washington.

JHS (Jackson Heart Study): The Jackson Heart Study (JHS) is supported and conducted in collaboration with Jackson State University (HHSN268201800013I), Tougaloo College (HHSN268201800014I), the Mississippi State Department of Health (HHSN268201800015I) and the University of Mississippi Medical Center (HHSN268201800010I, HHSN268201800011I and HHSN268201800012I) contracts from the National Heart, Lung, and Blood Institute (NHLBI) and the National Institute on Minority Health and Health Disparities (NIMHD). The authors also wish to thank the staffs and participants of the JHS.

KORA (Cooperative Health Research in the Augsburg Region): The KORA study was initiated and financed by the Helmholtz Zentrum München – German Research Center for Environmental Health, which is funded by the German Federal Ministry of Education and Research (BMBF) and by the State of Bavaria. Furthermore, KORA is funded by the Bavarian State Ministry of Health and Care through the research project DigiMed Bayern (www.digimed-bayern.de).**LBC1936 (Lothian Birth Cohort 1936):** We thank the LBC1936 cohort participants and team members who contributed to these studies. Phenotype collection was supported by Age UK (The Disconnected Mind project). Genotyping was funded by the BBSRC (BB/F019394/1). The work was undertaken by The University of Edinburgh Centre for Cognitive Ageing and Cognitive Epidemiology, part of the cross council Lifelong Health and Wellbeing Initiative (MR/K026992/1). Funding from the BBSRC and Medical Research Council (MRC) is gratefully acknowledged. We gratefully acknowledge the contribution of Lothian Birth Cohort co-author Professor John M. Starr, who died prior to the publication of this manuscript.

Lifelines (Netherlands Biobank): The Lifelines initiative has been made possible by subsidy from the Dutch Ministry of Health, Welfare and Sport, the Dutch Ministry of Economic Affairs, the University Medical Center Groningen (UMCG), Groningen University and the Provinces in the North of the Netherlands (Drenthe, Friesland, Groningen). The generation and management of GWAS genotype data for the Lifelines Cohort Study is supported by the Netherlands Organization of Scientific Research NWO (grant 175.010.2007.006), the Economic Structure Enhancing Fund (FES) of the Dutch government, the Ministry of Economic Affairs, the Ministry of Education,

Culture and Science, the Ministry for Health, Welfare and Sports, the Northern Netherlands Collaboration of Provinces (SNN), the Province of Groningen, University Medical Center Groningen, the University of Groningen, Dutch Kidney Foundation and Dutch Diabetes Research Foundation.

The authors wish to acknowledge the services of the Lifelines Cohort Study, the contributing research centers delivering data to Lifelines, and all the study participants.

MESA (Multi-Ethic Study of Atherosclerosis) and the **MESA SHARe** are conducted and supported by the National Heart, Lung, and Blood Institute (NHLBI) in collaboration with MESA investigators. Support for MESA is provided by contracts 75N92020D00001, HHSN268201500003I, N01-HC-95159, 75N92020D00005, N01-HC-95160, 75N92020D00002, N01-HC-95161, 75N92020D00003, N01-HC-95162, 75N92020D00006, N01-HC-95163, 75N92020D00004, N01-HC-95164, 75N92020D00007, N01-HC-95165, N01-HC-95166, N01-HC-95167, N01-HC-95168, N01-HC-95169, UL1-TR-000040, UL1-TR-001079, and UL1-TR-001420, UL1TR001881, DK063491, and R01HL105756. Funding for SHARe genotyping was provided by NHLBI Contract N02-HL-64278. Genotyping was performed at Affymetrix (Santa Clara, California, USA) and the Broad Institute of Harvard and MIT (Boston, Massachusetts, USA) using the Affymetrix Genome-Wide Human SNP Array 6.0. The authors thank the other investigators, the staff, and the participants of the MESA study for their valuable contributions. A full list of participating MESA investigators and institutes can be found at http://www.mesa-nhlbi.org.

NEO (The Netherlands Epidemiology of Obesity study): The authors of the NEO study thank all individuals who participated in the Netherlands Epidemiology in Obesity study, all participating general practitioners for inviting eligible participants and all research nurses for collection of the data. We thank the NEO study group, Petra Noordijk, Pat van Beelen and Ingeborg de Jonge for the coordination, lab and data management of the NEO study. The genotyping in the NEO study was supported by the Centre National de Génotypage (Paris, France), headed by Jean-Francois Deleuze. The NEO study is supported by the participating Departments, the Division and the Board of Directors of the Leiden University Medical Center, and by the Leiden University, Research Profile Area Vascular and Regenerative Medicine. Dennis Mook-Kanamori is supported by Dutch Science Organization (ZonMW-VENI Grant 916.14.023).

NESDA (Netherlands Study of Depression and Anxiety): Funding was obtained from the Netherlands Organization for Scientific Research (Geestkracht program grant 10-000-1002); the Center for Medical Systems Biology (CSMB, NWO Genomics), Biobanking and Biomolecular Resources Research Infrastructure (BBMRI-NL), VU University's Institutes for Health and Care Research (EMGO+) and Neuroscience Campus Amsterdam, University Medical Center Groningen, Leiden University Medical Center, National Institutes of Health (NIH, R01D0042157-01A, MH081802, Grand Opportunity grants 1RC2 MH089951 and 1RC2 MH089995). Part of the genotyping and analyses were funded by the Genetic Association Information Network (GAIN) of the Foundation for the National Institutes of Health.Computing was supported by BiG Grid, the Dutch e-Science Grid, which is financially supported by NWO.

Pelotas Birth Cohort Study (The 1982 Pelotas Birth Cohort Study, Brazil): The 1982 Pelotas Birth Cohort Study is conducted by the Postgraduate Program in Epidemiology at Universidade Federal de Pelotas with the collaboration of the Brazilian Public Health Association (ABRASCO). From 2004 to 2013, the Wellcome Trust supported the study. The International Development

Research Center, World Health Organization, Overseas Development Administration, European Union, National Support Program for Centers of Excellence (PRONEX), the Brazilian National Research Council (CNPq), and the Brazilian Ministry of Health supported previous phases of the study.

Genotyping of 1982 Pelotas Birth Cohort Study participants was supported by the Department of Science and Technology (DECIT, Ministry of Health) and National Fund for Scientific and Technological Development (FNDCT, Ministry of Science and Technology), Funding of Studies and Projects (FINEP, Ministry of Science and Technology, Brazil), Coordination of Improvement of Higher Education Personnel (CAPES, Ministry of Education, Brazil).

RS (Rotterdam Study): The Rotterdam Study is supported by the Erasmus MC University Medical Center and Erasmus University Rotterdam; The Netherlands Organisation for Scientific Research (NWO); The Netherlands Organisation for Health Research and Development (ZonMw); the Research Institute for Diseases in the Elderly (RIDE); The Netherlands Genomics Initiative (NGI); the Ministry of Education, Culture and Science; the Ministry of Health, Welfare and Sports; the European Commission (DG XII); and the Municipality of Rotterdam. The contribution of inhabitants, general practitioners and pharmacists of the Ommoord district to the Rotterdam Study is gratefully acknowledged. The contributions to the Management Team specifically and the Rotterdam Study at large of the following persons are pivotal to the daily operations of the study and highly appreciated: Jacobus Lubbe, Gabriëlle Bakker, Eric Neeleman, Jeannette Vergeer, Anneke Korving, Pauline van Wijngaarden, Jolande Verkroost – van Heemst, Silvan Licher, and Isabel van der Velpen.

The generation and management of GWAS genotype data for the Rotterdam Study was executed by the Human Genotyping Facility of the Genetic Laboratory of the Department of Internal Medicine, Erasmus MC, Rotterdam, The Netherlands. The GWAS datasets are supported by the Netherlands Organisation of Scientific Research NWO Investments (nr. 175.010.2005.011, 911-03-012), the Genetic Laboratory of the Department of Internal Medicine, Erasmus MC, the Research Institute for Diseases in the Elderly (014-93-015; RIDE2), the Netherlands Genomics Initiative (NGI)/Netherlands Organisation for Scientific Research (NWO) Netherlands Consortium for Healthy Aging (NCHA), project nr. 050-060-810. We thank Pascal Arp, Mila Jhamai, Marijn Verkerk, Lizbeth Herrera, Marjolein Peters and Carolina Medina-Gomez for their help in creating the GWAS database, and Karol Estrada, Yurii Aulchenko and Carolina Medina-Gomez for the creation and analysis of imputed data.

SP2 (Singapore Prospective Study Program): The SP2 study is supported by individual research and clinical scientist award schemes from the National Medical Research Council (NMRC) and the Biomedical Research Council (BMRC) of Singapore, the Singapore Ministry of Health, National University of Singapore and National University Health System, Singapore. The Genome Institute of Singapore, Agency for Science, Technology and Research, Singapore provided services for genotyping.

SHIP (Study of Health in Pomerania): SHIP is part of the Community Medicine Research net of the University of Greifswald, Germany, which is funded by the Federal Ministry of Education and Research (grants no. 01ZZ9603, 01ZZ0103, and 01ZZ0403), the Ministry of Cultural Affairs as well as the Social Ministry of the Federal State of Mecklenburg-West Pomerania, and the network 'Greifswald Approach to Individualized Medicine (GANI_MED)' funded by the Federal Ministry of Education and Research (grant 03IS2061A). Genome-wide data were supported by the Federal

Ministry of Education and Research (grant no. 03ZIK012) and a joint grant from Siemens Healthcare, Erlangen, Germany and the Federal State of Mecklenburg- West Pomerania. The University of Greifswald is a member of the 'Center of Knowledge Interchange' program of the Siemens AG.

SWHS/SMHS (Shanghai Women's Health Study/ Shanghai Men's Health Study): We thank all the individuals who took part in these studies and all the researchers who have enabled this work to be carried out. The Shanghai Women's Health Study and the Shanghai Men's Health Study are supported by research grants UM1CA182910 and UM1CA173640 from the U.S. National Cancer Institute, respectively.

TRAILS (TRacking Adolescents' Individual Lives Survey): TRAILS is a collaborative project involving various departments of the University Medical Center and University of Groningen, the University of Utrecht, the Radboud University Medical Center, and the Parnassia Psychiatric Institute, all in the Netherlands. TRAILS has been financially supported by grants from the Netherlands Organization for Scientific Research NWO (Medical Research Council program grant GB-MW 940-38-011; ZonMW Brainpower grant 100-001-004; ZonMw Risk Behavior and Dependence grant 60-60600-97-118; ZonMw Culture and Health grant 261-98-710; Social Sciences Council medium-sized investment grants GB-MaGW 480-01-006 and GB-MaGW 480-07-001; Social Sciences Council project grants GB-MaGW 452-04-314 and GB-MaGW 452-06-004; ZonMw Longitudinal Cohort Research on Early Detection and Treatment in Mental Health Care grant 636340002; NWO large-sized investment grant 175.010.2003.005; NWO Longitudinal Survey and Panel Funding 481-08-013 and 481-11-001; NWO Vici 016.130.002, 453-16-007/2735, and Vi.C.191.021; NWO Gravitation 024.001.003), the Dutch Ministry of Justice (WODC), the European Science Foundation (EuroSTRESS project FP-006), the European Research Council (ERC-2017-STG-757364 and ERC-CoG-2015-681466), Biobanking and Biomolecular Resources Research Infrastructure BBMRI-NL (CP 32), the participating universities, and Accare Centre for Child and Adolescent Psychiatry. Statistical analyses were carried out on the Genetic Cluster Computer (http://www.geneticcluster.org), which is financially supported by the Netherlands Scientific Organization (NWO 480-05-003) along with a supplement from the Dutch Brain Foundation.

We are grateful to everyone who participated in this research or who worked on this project to make it possible.
WHI (Women's Health Initiative): The WHI program is funded by the National Heart, Lung, and Blood Institute, National Institutes of Health, U.S. Department of Health and Human Services through contracts HHSN268201600018C, HHSN268201600001C, HHSN268201600002C, HHSN268201600003C, and HHSN268201600004C." The authors thank the WHI investigators and staff for their dedication, and the study participants for making the program possible. A full listing of WHI investigators can be found at: https://www-whi-org.s3.us-west-2.amazonaws.com/wp-content/uploads/WHI-Investigator-Long-List.pdf

YFS (The Cardiovascular Risk in Young Finns Study): The Young Finns Study has been financially supported by the Academy of Finland: grants 356405, 322098, 286284, 134309 (Eye), 126925, 121584, 124282, 129378 (Salve), 117797 (Gendi), and 141071 (Skidi); the Social Insurance Institution of Finland; Competitive State Research Financing of the Expert Responsibility area of Kuopio, Tampere and Turku University Hospitals (grant X51001); Juho Vainio Foundation; Paavo Nurmi Foundation; Finnish Foundation for Cardiovascular Research ;

Finnish Cultural Foundation; The Sigrid Juselius Foundation; Tampere Tuberculosis Foundation; Emil Aaltonen Foundation; Yrjö Jahnsson Foundation; Signe and Ane Gyllenberg Foundation; Diabetes Research Foundation of Finnish Diabetes Association; EU Horizon 2020 (grant 755320 for TAXINOMISIS and grant 848146 for To Aition); European Research Council (grant 742927 for MULTIEPIGEN project); Tampere University Hospital Supporting Foundation, Finnish Society of Clinical Chemistry, the Cancer Foundation Finland, and pBETTER4U_EU (Preventing obesity through Biologically and bEhaviorally Tailored inTERventions for you; project number: 101080117).

We thank the teams that collected data at all measurement time points; the persons who participated as both children and adults in these longitudinal studies; and biostatisticians Irina Lisinen, Johanna Ikonen, Noora Kartiosuo, Ville Aalto, and Jarno Kankaanranta for data management and statistical advice.
